# Engineering Efferocytosis‐Mimicking Nanovesicles to Regulate Joint Anti‐Inflammation and Peripheral Immunosuppression for Rheumatoid Arthritis Therapy

**DOI:** 10.1002/advs.202404198

**Published:** 2024-05-29

**Authors:** Shanshan Yuan, Yingqian Chai, Jianghua Xu, Youchao Wang, Lihua Jiang, Ning Lu, Hongyi Jiang, Jilong Wang, Xiaoyun Pan, Junjie Deng

**Affiliations:** ^1^ Joint Centre of Translational Medicine The First Affiliated Hospital of Wenzhou Medical University Wenzhou Zhejiang 325000 China; ^2^ Joint Centre of Translational Medicine Wenzhou Institute University of Chinese Academy of Sciences Wenzhou Zhejiang 325000 China; ^3^ Zhejiang Engineering Research Center for Tissue Repair Materials Wenzhou Institute University of Chinese Academy of Sciences Wenzhou Zhejiang 325000 China; ^4^ Chimie ParisTech PSL University CNRS Institute of Chemistry for Life and Health Sciences Laboratory for Inorganic Chemical Biology Paris 75005 France; ^5^ Department of Orthopaedics The Second Affiliated Hospital and Yuying Children's Hospital of Wenzhou Medical University Wenzhou 325000 China

**Keywords:** efferocytosis‐mimicking, joint anti‐inflammation, macrophage polarization, peripheral immunosuppression, rheumatoid arthritis, Treg differentiation

## Abstract

Rheumatoid arthritis (RA) is an autoimmune disorder characterized by chronic inflammation of the synovial joints and the dysfunction of regulatory T cells (Tregs) in the peripheral blood. Therefore, an optimal treatment strategy should aim to eliminate the inflammatory response in the joints and simultaneously restore the immune tolerance of Tregs in peripheral blood. Accordingly, we developed an efferocytosis‐mimicking nanovesicle that contains three functional factors for immunomodulating of efferocytosis, including “find me” and “eat me” signals for professional (macrophage) or non‐professional phagocytes (T lymphocyte), and “apoptotic metabolite” for metabolite digestion. We showed that efferocytosis‐mimicking nanovesicles targeted the inflamed joints and spleen of mice with collagen‐induced arthritis, further recruiting and selectively binding to macrophages and T lymphocytes to induce M2 macrophage polarization and Treg differentiation and T helper cell 17 (Th17) recession. Under systemic administration, the efferocytosis‐mimicking nanovesicles effectively maintained the pro‐inflammatory M1/anti‐inflammatory M2 macrophage balance in joints and the Treg/Th17 imbalance in peripheral blood to prevent RA progression. This study demonstrates the potential of efferocytosis‐mimicking nanovesicles for RA immunotherapy.

## Graphical abstract

We designed and prepared efferocytosis‐mimicking nanovesicles, that can effectively target inflamed joints, induce anti‐inflammatory M2 macrophages, and accumulate in the spleen to regulate the imbalance between Treg and Th17 cells, thereby promoting peripheral immunosuppression and suppressing RA progression.

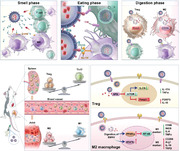

## 1 Introduction

Rheumatoid arthritis (RA) is a chronic autoimmune disease characterized by joint and systemic inflammation.^[^ ^1^, ^2^, ^3^ ^]^ The dysfunction of regulatory T cells (Tregs) plays a crucial role in the breakdown of self‐tolerance,^[^ ^4^, ^5^ ^]^ leading to a proliferative autoimmune disorder within the peripheral blood and the infiltration of inflammatory immune cells (including proinflammatory M1 macrophages^[^ ^6^, ^7^ ^]^ and pathogenic CD4^+^ T cells^[^ ^8^, ^9^ ^]^) into the synovial membrane. These factors contribute jointly to the development of chronic joint synovitis and systemic immune complications.^[^ ^10^, ^11^ ^]^ Traditional therapeutic approaches have primarily focused on suppressing the inflammatory processes in inflamed joints through various means, such as inhibiting inflammatory cytokines,^[^ ^12^, ^13^ ^]^ depleting M1 macrophages, and inducing a shift in the proinflammatory M1 to anti‐inflammatory M2 macrophage phenotype.^[^ ^14^, ^15^, ^16^, ^17^, ^18^, ^19^ ^]^ Nanocarriers incorporating anti‐inflammatory agents have emerged as method for selectively targeting inflamed joints and promoting M2 macrophages polarization, however, their long‐term efficacy in preventing inflammation and T cells‐mediated RA progression is limited.^[^ ^2^, ^20^, ^21^ ^]^ This is primarily attributed to an imbalance between Treg and T helper 17 (Th17) cells, characterized by a decrease and increase in Treg and Th17 levels, respectively, in both lymphoid tissues and peripheral blood.^[^ ^22^, ^23^, ^24^ ^]^ This imbalance promotes in the production of inflammatory cytokines, such as interleukin‐17 (IL‐17) and tumor necrosis factor (TNF‐α), which perpetuate the infiltration of M1 macrophages into synovial tissues, contributing to synovitis.^[^ ^21^, ^23^, ^25^ ^]^ Consequently, there is an urgent need to develop novel approaches that not only target and inhibit joint inflammation but also enhance the levels of Tregs for systemic immunosuppression to effectively prevent the progression of RA.

Efferocytosis, an active biochemical process that clears apoptotic cells via phagocytes, has anti‐inflammatory effects and occurs at distinct stages.^[^ ^26^, ^27^ ^]^ Firstly, apoptotic cells release “find me” signals, such as sphingosine (S1P), to attract phagocytes (primarily macrophages); secondly, the expression of “eat me” signals, such as phosphoserine (PS), on the surface of apoptotic cells allows them to be recognized and engulfed by phagocytes; finally, apoptotic cells digestion stimulates phagocytes to secrete anti‐inflammatory cytokines, including interleukin‐10 (IL‐10) and transforming growth factor‐β (TGF‐β).^[^ ^28^, ^29^, ^30^, ^31^ ^]^ Additionally, the intravenous infusion of apoptotic cells primarily results in their elimination within the liver and spleen, which can induce a phenotypic switch in spleenic T lymphocytes towards a Treg phenotype.^[^ ^32^, ^33^, ^34^ ^]^ Therefore, efferocytosis has a profound impact on both innate and adaptive immunity.^[^ ^35^ ^]^ Based on the distinct immunomodulatory properties of efferocytosis, we hypothesized that nanovesicles combining “find me” signals, “eat me” signals, and apoptosis‐related agents can effectively target the inflamed joints to induce M2 macrophages and accumulate in the spleen to regulate the Treg/Th17 imbalance, thereby promoting peripheral immunosuppression and suppressing RA progression. Some studies have reported that using PS‐modified nanocarriers as phagocytic signals can effectively target and inhibit M1 macrophages in RA, atherosclerosis, and obesity.^[^ ^36^, ^37^, ^38^ ^]^ Notably, merely incorporating “find me” signals is insufficient to fully emulate the function of efferocytosis. Both the “find me” and “digestion” phases are crucial in recruiting phagocytes and maintaining metabolic homeostasis. Consequently, the use of nanocarriers that mimic efferocytosis for the treatment of RA has not yet been reported, and their potential in this context remains poorly understood.

**Scheme 1:**
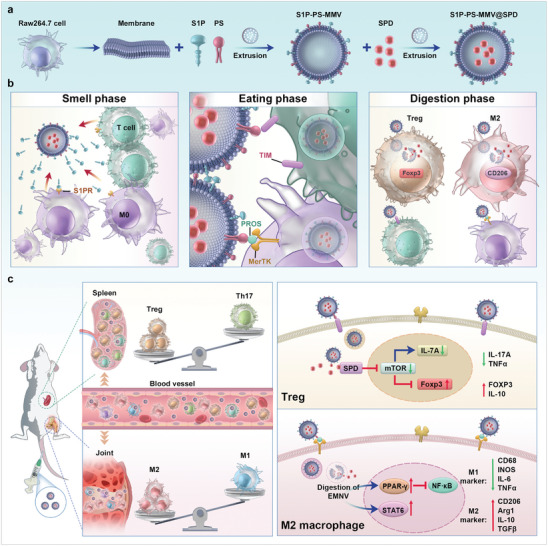
Synthesis and immune‐regulating mechanisms of efferocytosis‐mimicking nanovesicle (EMNV, S1P‐PS‐MMV@SPD). a) Schematic illustration of EMNV fabrication and b)interaction of EMNV with professional (macrophage) or non‐professional phagocyte (T lymphocyte) during three distinct phases: “smell phase,” “eating phase,” and “digestion phase.” c) Mechanistic explanation for the specific regulation of macrophages and T cells by EMNVs. The therapeutic efficacy of EMNVs against RA are associated with their targeting of the inflamed joint to induce M2 macrophages and accumulation in the spleen to increase Treg levels.

This study investigated the performance of an efferocytosis‐mimicking nanovesicle (EMNV or S1P‐PS‐MMV@SPD) that expresses S1P (find me signal) and surface PS (eat me signal) and is loaded with spermidine (SPD, an apoptotic metabolite) within the core of a macrophage membrane‐derived vesicle (MMV) for alleviating joint inflammation and enhancing peripheral immunosuppression in RA treatment (**Scheme** **1**). After intravenous injection administration, nanovesicles can accumulate within the inflamed joints and spleen through the inflammation‐targeting ability of macrophage membranes.^[^ ^39^ ^]^ Meanwhile, the modification of nanovesicles with S1P and PS enables them to actively recruit and selectively bind to macrophages and T lymphocytes‐cells that overexpress PS receptors on their surfaces.^[^ ^40^, ^41^ ^]^ Upon internalization, SPD, a metabolite secreted by apoptotic cells,^[^ ^42^ ^]^ is gradually released from the nanovesicles to mitigate the inflammatory response, induce M2 macrophage polarization and enhane Treg differentiation.^[^ ^43^, ^44^ ^]^ Consequently, in a collagen‐induced arthritis (CIA) mouse model, we showed that the administration of S1P‐PS‐MMV@SPD induced M2 polarization and increased Treg levels, while reducing the Th17 cell fraction, thereby attenuating RA progression. Our study highlights the effectiveness of this innovative nanovesicular tactic to simultaneously reduce joint inflammation and restore peripheral immunosuppression by mimicking efferocytosis in RA immunotherapy.

## 2 Results and Discussions

### 2.1 Preparation and Characterization of EMNV

**Figure 1:**
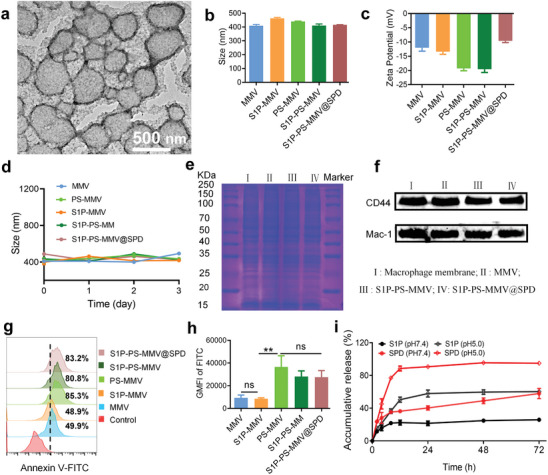
Characterization of EMNV. a) Representative TEM image of S1P‐PS‐MMV@SPD. Size b) and zeta potentials c) of vesicles. d) Stability of vesicles. e) Protein bands of macrophage membranes, MMV, S1P‐PS‐MMV, and S1P‐PS‐MMV@SPD based on SDS‐PAGE. f) Representative proteins (CD44 and Mac‐1) in macrophage membranes, MMV, SIP‐PS‐MMV, and S1P‐PS‐MMV@SPD were determined using western blot assays. g) Flow cytometry histogram of phosphatidylserine presentation, and h) quantification of PS (FITC‐AnnexinV^+^). i) Release profile of S1P and SPD from S1P‐PS‐MMV@SPD under different pH conditions. Data are expressed as mean ± SD (n = 3). ^**^ *p* < 0.01, ns = no significance.

The procedure for preparing S1P‐PS‐MMV@SPD is illustrated in Scheme 1. S1P and PS were inserted into the membranes of macrophage membrane‐derived vesicles (MMVs) and SPD was encapsulated inside the MMV through a co‐extrusion approach. Transmission electron microscopy (TEM) imaging showted a spheroid micromorphology for S1P‐PS‐MMV@SPD (**Figure** **1**a). Dynamic light scattering analysis suggested that the average diameter of the S1P‐PS‐MMV@SPD was ∼416.4 ± 1.9 nm, similar to that of natural apoptotic bodies (100 ∼5000 nm),^[^ ^45^ ^]^ and the polydispersity index was ∼0.25 (Figures 1b and S1). After the insertion of S1P and PS, the zeta potential of the S1P‐PS‐MMV decreased to −19.6 ± 1.1 mV (Figure 1c), primarily due to the negative charge of S1P and PS. Meanwhile, the zeta potential of the S1P‐PS‐MMV@SPD (−9.6 ± 0.6 mV) was greater than that of the S1P‐PS‐MMV, suggesting that the positively charged SPD was successfully loaded inside the S1P‐PS‐MMV. All vesicles exhibited excellent stability in phosphate‐buffered saline (PBS) containing 10% serum for 3 d (Figure 1d). Sodium dodecyl sulfate‐polyacrylamide gel electrophoresis (SDS‐PAGE) showed that the protein components of the EMNV and macrophage membranes were similar (Figure 1e). Western blot analysis also confirmed that the MMV, S1P‐PS‐MMV, and S1P‐PS‐MMV@SPD retained the key functional proteins (CD44 and Mac‐1) with inflammatory targeting properties from macrophage membranes (Figure 1f).^[^ ^46^ ^]^ We confirmed the presence of PS on the surface of the S1P‐PS‐MMV@SPD using an Annexin V apoptosis kit (Figure 1g,h). The concentrations of S1P and SPD in the EMNV and their in vitro release performance were examined in PBS (pH = 7.4 and 5.0) at room temperature (Figure 1i). The standard curve and mass spectrum analyses (500 ng mL^−1^) of S1P and SPD were performed using liquid chromatography‐mass spectrometry (LC‐MS) (Figure S2). and high‐performance liquid chromatography (HPLC), respectively (Figure S3). The S1P‐PS‐MMV@SPD containing 16.8 µg membrane protein could load 1.15 ± 0.08 µg S1P and 9.94 ± 0.50 µg SPD simultaneously. On day 3, under physiological conditions (pH 7.4), the accumulative release of S1P and SPD reached 24.17 ± 1.67% and 57.89 ± 6.16%, respectively. Meanwhile, the rate and amount released by the EMNV were greater under acidic conditions (pH 5.0) than under physiological conditions. These findings demonstrated that the EMNV has the expected properties of apoptotic bodies, releasing recruitment signals, containing phagocytosis signals, and releasing apoptotic metabolites.

To assess potential toxicity, free SPD and vesicles were incubated with three different kinds of cell lines: L929 cells, bone marrow‐derived macrophages (BMDMs) and T lymphocytes. The positivity rate of F4/80 in BMDMs was 98%, and the purity of T lymphocytes after sorting reached 83.9% (Figures S4 and S5). SPD (50 µM) exhibited no appreciable toxicity in any cell line (Figure S6). Additionally, no obvious effect on cell viability was observed for any of the vesicles, indicating the excellent biocompatibility of the EMNV (Figure S7). Overall, the EMNV was successfully prepared for efferocytosis, i.e., to release of the recruitment signal (S1P), express of PS for recognition by phagocytes, and gradually release representatively apoptotic metabolites that exhibit immunomodulatory effects.

### 2.2 Efferocytosis of EMNV by Macrophages in vitro

**Figure 2:**
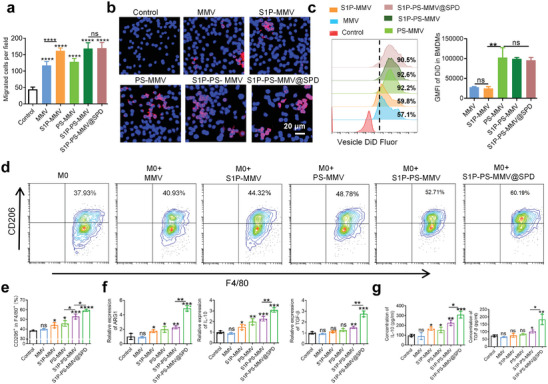
Efferocytosis of EMNVs by macrophages in vitro. a) Quantification of migrated BMDMs. b) Intracellular uptake of vesicles by BMDMs after incubation for 2 h. c) Flow cytometry and geometric median fluorescence intensity (GMFI) of DiD signals in BMDMs after incubation with DiD‐labeled vesicles for 2 h. Vesicles were labeled with DiD (red) and BMDMs nuclei were labeled with 4′,6‐diamidino‐2‐phenylindole (DAPI, blue). The scale bar in the bottom‐right image applies for all images. d) Representative flow cytometric chart of M0 macrophages after different treatments and e) quantification of CD206 expression. f) Relative mRNA expression (ARG1, IL‐10, and TGF‐β) in M0 macrophages after different treatments. g) Concentrations of IL‐10 and TGF‐β in culture media of M0 macrophages after different treatments. Data are expressed as mean ± SD (n = 3). ^*^ *p* < 0.05, ^**^ *p* < 0.01, ^***^ *p* < 0.005, ^****^ *p* < 0.0001, ns = no significance.

During efferocytosis, macrophages are a prevalent type of professional phagocytes that engulf and clear nearby apoptotic cells. Macrophages are recruited by “find me” signals and engulf the recognized apoptotic cells after interaction between the “eat me” signals and receptors on the macrophage surface. Finally, the anti‐inflammatory response of the macrophages is elicited through the secretion of anti‐inflammatory cytokines.^[^ ^26^, ^27^ ^]^ To examine the effect of EMNVs on macrophage recruitment, a transwell migration model was used to assess the migration of M0 macrophages (naive BMDMs). Both the MMV and PS‐MMV induced the migration of BMDMs (**Figure** **2**a and S8), which could be attributed to the inherent S1P expression in the MMV membrane.^[^ ^47^ ^]^ Moreover, the average number of migrating BMDMs after S1P‐MMV, S1P‐PS‐MMV, and S1P‐PS‐MMV@SPD treatments were significantly higher than those after MMV or PS‐MMV treatments, suggesting that S1P modification could effectively recruit BMDMs.

During efferocytosis, exposure of PS on the surface of apoptotic cells allows them to be rapidly removed by macrophages, thus preventing the “explosion” of apoptotic cells and release of proinflammatory factors.^[^ ^48^ ^]^ The uptake efficiency of the EMNV in BMDMs was monitored using1,1′‐dioctadecyl‐3,3,3′,3′‐tetramethylindodicarbocyanine,4‐chlorobenzenesulfonate salt (DiD) as a fluorescence indicator (Figure 2b). Confocal laser scanning microscopy (CLSM) images illustrated that the PS‐MMV, S1P‐PS‐MMV, and S1P‐PS‐MMV@SPD had stronger fluorescence signals than the MMV and S1P‐MMV. Flow cytometry was performed to quantify vesicle phagocytosis. The geometric mean fluorescence intensities (GMFI) of the MMV‐PS, MMV‐S1P‐PS, and MMV‐S1P‐PS@SPD in BMDMs were ∼3.5 times higher than those of the MMV and S1P‐MMV (Figure 2c), indicating that PS insertion promotes the ability of recognition and phagocytosis by macrophages.

We explored the immunomodulation of macrophages by EMNV, after co‐incubating BMDMs with EMNVs for 24 h. Compared to S1P‐MMV and PS‐MMV treatments, the S1P‐PS‐MMV treatment showed a noticeable increase in the percentage of F4/80^+^CD206^+^ M2 macrophages (Figure 2d,e). The S1P‐PS‐MMV@SPD treatment resulted in the highest proportion of M2 macrophages. Both S1P‐MMV and PS‐MMV treatment slightly enhanced the mRNA expression and production of anti‐inflammatory signals (such as arginase 1 (ARG1) and IL‐10) in BMDMs (Figure 2f,g). In contrast, S1P‐PS‐MMV and S1P‐PS‐MMV@SPD treatments significantly increased the mRNA expression of ARG1, IL‐10, and TGF‐β, as well as the production of IL‐10 and TGF‐β. Notably, the S1P‐PS‐MMV@SPD exhibited the most pronounced anti‐inflammatory effect. These findings suggested that the S1P‐PS‐MMV@SPD promotes the transformation of macrophages towards an anti‐inflammatory M2 phenotype. Considering their excellent recruitment and rapid recognition properties, the S1P‐PS‐MMV and S1P‐PS‐MMV@SPD were selected for the subsequent experiments.

**Figure 3:**
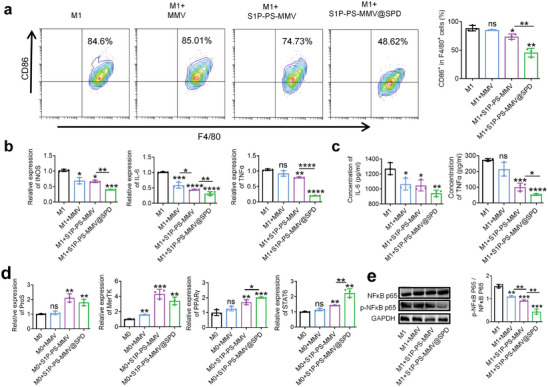
Regulation of macrophages by EMNV in vitro. a) Representative flow cytometric chart of M1 macrophages after different treatments and quantification analyses of CD86 expression. b) Relative mRNA expression (INOS, IL‐6, and TNF‐α) in M1 macrophages after different treatments. c) Concentrations of IL‐6 and TNF‐α in culture media of M1 macrophages after different treatments. d) Relative mRNA expression (MerTK, ProS, PPARγ, and STAT6 in M0 macrophages after different treatments. e) Protein expression of NF‐κB p65 and p‐NF‐κB p65 detected by western blot analysis using specific antibodies. Data are expressed as mean ± SD (n = 3). ^*^ *p* < 0.05, ^**^ *p* < 0.01, ^***^ *p* < 0.005, ^****^ *p* < 0.0001, ns = no significance.

In RA, numerous inflammatory M1 macrophages infiltrate the synovial tissue and secrete proinflammatory cytokines involved in cartilage injury.^[^ ^49^ ^]^ We further studied the effect of the EMNV on inflammatory M1 macrophages. The S1P‐PS‐MMV@SPD reduced the average percentage of F4/80^+^CD86^+^ M1 macrophages from 88.57 ± 4.78% to 45.52 ± 4.82% (**Figure** **3**a). Compared to MMV and S1P‐PS‐MMV treatments, the S1P‐PS‐MMV@SPD treatment decreased the pro‐inflammation‐related mRNA expressions of inducible nitric oxide synthase (INOS), interleukin‐6 (IL‐6), and TNF‐α in M1 macrophages and the secretion of TNF‐α and IL‐6 (Figure 3b,c). The inhibitory effect of the S1P‐PS‐MMV@SPD on M1 macrophages was primarily attributed to the macrophage membrane and SPD. Previous studies have reported that macrophage membranes can reduce proinflammatory cytokines levels by binding and neutralizing cytokines.^[^ ^50^ ^]^ Furthermore, SPD can exert anti‐inflammatory effects on macrophages by suppressing the nuclear factor kappa B (NF‐κB) pathway.^[^ ^51^ ^]^

The clearance of apoptotic cells by macrophages is benefited by the efficient interaction between PS exposed on apoptotic cells and receptors on the macrophage membrane.^[^ ^52^ ^]^ Therefore, we investigated the mRNA expressions of PS recognition receptors [Mer receptor tyrosine kinase (MerTK) and Protein S (ProS)] in BMDMs after co‐incubation with vesicles for 24 h using quantitative real‐time polymerase chain reaction (qRT‐PCR). After S1P‐PS‐MMV and S1P‐PS‐MMV@SPD treatments, the expression levels of PS receptors (MerTK and ProS) in BMDMs were significantly elevated (Figure 3d). The interaction between macrophages and apoptotic cells activates peroxisome proliferator‐activated receptor gamma (PPARγ), which controlled the expression of PS recognition receptors.^[^ ^53^ ^]^ This, in turn, accelerates the clearance of apoptotic cells and triggeres the expression of Transducer and Activator of Transcription 6 (STAT6) in a feed‐forward manner, promoting M2 activation.^[^ ^54^ ^]^ Moreover, PPARγ can reduce inflammation levels by controlling NF‐κB.^[^ ^55^ ^]^ To further investigate whether anti‐inflammation‐inducing mechanism of EMNV is similar to that of natural apoptotic cells, we examined the mRNA expression levels of STAT6 and PPARγ using qRT‐PCR, as well as the protein expression level of NF‐κB through western blot analysis. The S1P‐PS‐MMV@SPD promoted the expression of PPARγ and STAT6 while suppressing NF‐κB mediated inflammation levels (Figure 2d,e). These findings suggested that the S1P‐PS‐MMV@SPD could promote the anti‐inflammatory effect of M0 macrophages and alleviate the levels of pro‐inflammatory M1 macrophages, indicating an immunomodulatory effect on macrophages as a potential RA treatment.

### 2.3 Efferocytosis of EMNV by T‐lymphocytes in vitro

During efferocytosis, T cells are typically considered unable to recognize and engulf apoptotic cells in the same manner as professional phagocytes. However, T cells may participate in the S1P‐induced “smell phase” and PS‐mediated “eating phase.” Baeyens et al.^[^ ^56^ ^]^ reported that T cells were more prone to migrate towards high concentrations of S1P and exhibited an extended residence time. Wang et al.^[^ ^57^ ^]^ confirmed that T cells also recognized PS directly through the expression of T cell immunoglobulin mucin (TIM) proteins, leading to the suppression of Th17 differentiation and the preservation of Tregs. Therefore, we hypothesized that EMNVs could recruit and recognize T cells to perform immune functions.

**Figure 4:**
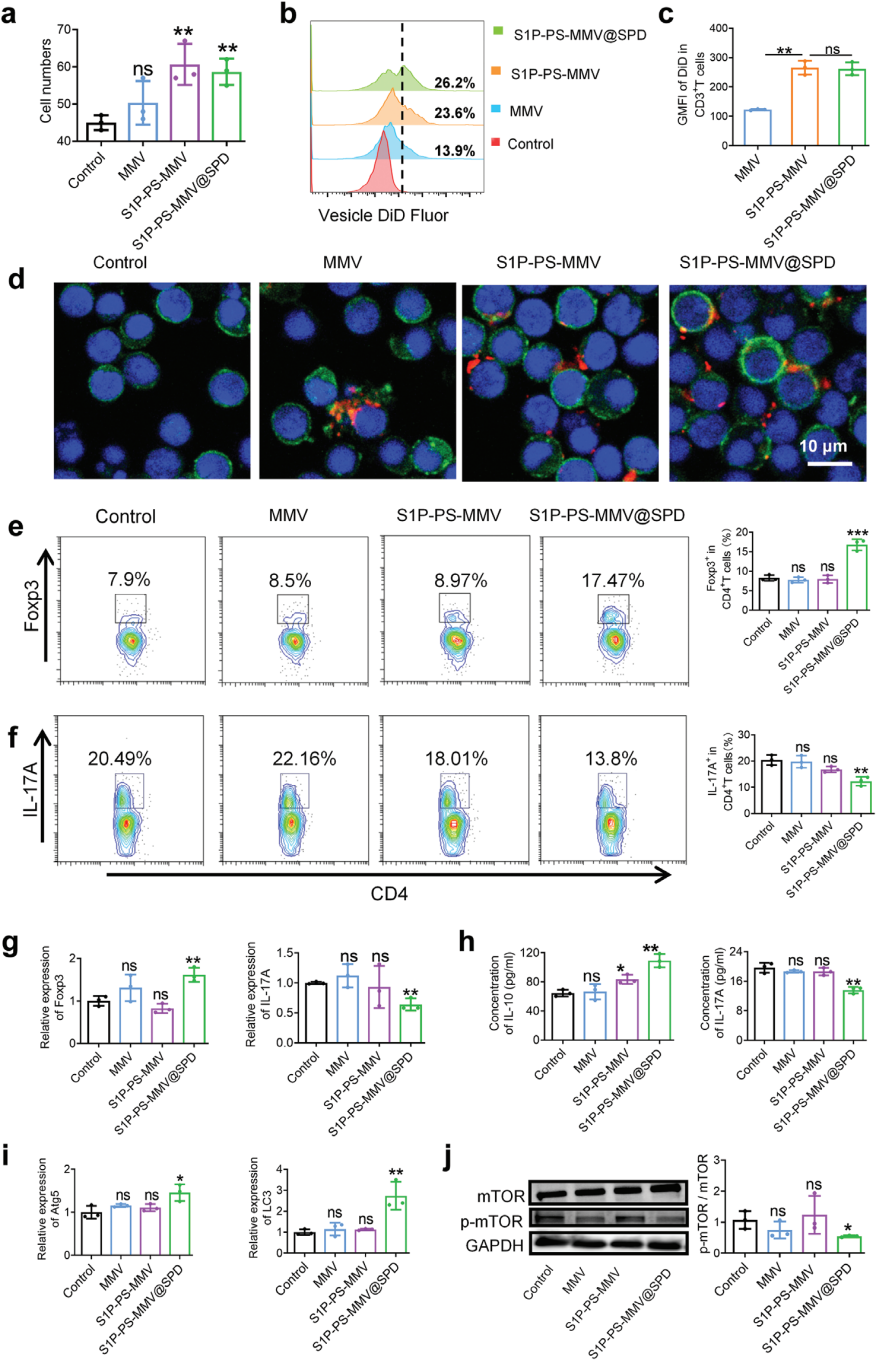
Efferocytosis of EMNVs by T lymphocytes in vitro. a) Quantification analyses of migrated T cells. b) Flow cytometry analysis and c) geometric median fluorescence intensity (GMFI) of DiD signals in T cells after incubation with DiD‐labeled vesicle for 24 h. d) Confocal microscopy image of intracellular uptake of vesicles by T cells from spleen after incubation for 24 h. Vesicles were labeled with DiD (red), T cells were labeled with anti‐CD3 (green), and nuclei of cells were labeled with DAPI (blue). The scale bar in the far‐right image applies to all the images. e) Representative flow cytometric plot and correspondingly quantification analyses of CD3^+^CD4^+^Foxp3^+^ Tregs. f) Flow cytometric plot and correspondingly quantities of CD4^+^IL‐17A^+^ T cells. g) Relative mRNA expression of Foxp3 and IL‐17A in T cells after different treatments. h) Concentrations of IL‐10 and IL‐17A in supernatant of T cells after different treatments. i) Relative mRNA expression of Atg5 and LC3 in T cells after different treatments. j) Expression of mTOR and p‐mTOR were detected using western blot analysis. In these experiments (Figure 3e–j), T cells were cultured under Th17 polarization conditions. Data are expressed as mean ± SD (n = 3). ^*^ *p* < 0.05, ^**^ *p* < 0.01, ns = no significance.

To determine whether T cells also engage in efferocytosis‐like interactions with macrophage‐like vesicles, we also evaluated the migration and recognition of EMNVs by T cells from the spleen. Vesicles containing S1P enhanced T cells migration (**Figure** **4**a). There was no change in the uptake of PS‐modified vesicles by T cells after co‐culturing for 2 h (Figure S9). However, after 24 h, T cells showed enhanced phagocytic efficiency when co‐cultured with PS‐modified vesicles (Figure 4b,c). The CLSM imaging and flow cytometry results were consistent (Figure 4d). Previous studies reported that the Treg/Th17 cell imbalance may promote RA progression. Therefore, it is especially important to regulate T‐cell responses while controlling inflammation to effectively manage the disease.^[^ ^22^ ^]^ Consequently, we assessed whether Treg modulation could be achieved via the direct interaction of vesicles with T cells. A gating strategy diagram of the T cells is shown in Figure S10. After incubating T cells isolated from the spleen with vesicles for 24 h, the proliferation of CD3^+^CD4^+^ T cells was suppressed (Figure S11). The effects of the S1P‐PS‐MMV@SPD on Th17 and Treg cell differentiation were analyzed using flow cytometry. Compared to the control group, the percentage of CD3^+^CD4^+^Foxp3^+^ Tregs after the S1P‐PS‐MMV@SPD treatment increased significantly from 8.28 ± 0.73% to 16.79 ± 1.43% (Figure 4e). To investigate the effect of EMNVs on Th17 cells differentiation, we differentiated T cells under Th17 polarization conditions in the presence of EMNVs. Figure 3e illustrated a noticeable decrease in the percentage of CD3^+^CD4^+^IL‐17A^+^ T cells from 20.4 ± 1.95% to 12.28 ± 1.69%. Compared to the other groups, there was an increase in the mRNA levels of Treg‐related genes (Foxp3) and decrease in Th17‐related genes such as IL‐17A after the S1P‐PS‐MMV@SPD treatment (Figure 4g). The concentration of IL‐10 in the culture medium of T cells obviously increased after the S1P‐PS‐MMV@SPD treatment, whereas the concentration of IL‐17A decreased (Figure 4h). Notably, the S1P‐PS‐MMV without SPD promoted migration and engulfment, but had little effect on the regulation of T cells. PS primarily mediates the interaction between apoptotic bodies and T cells during the in eating phase. During the digestion phase, the apoptotic metabolites (such as SPD) released from apoptotic cells play the primary role in regulating T cell responses.^[^ ^57^ ^]^ The immunomodulatory effect of SPD on T cells is mediated by an intact autophagy, which is regulated by mechanistic target of rapamycin kinase (mTOR, a crucial regulator of T cell differentiation and function). Indeed, mTOR inhibition is closely associated with the induction of CD3^+^CD4^+^Foxp3^+^ Tregs.^[^ ^43^ ^]^ To further investigate this mechanism of T cells regulation by EMNV, we examined the mRNA expression levels of autophagy‐related gene 5 (Atg5) and microtubule‐associated protein 1 light chain 3 (LC3) using qRT‐PCR, as well as the levels of mTOR through western blot analysis. The S1P‐PS‐MMV@SPD accelerated Atg5 and LC3 expression and decreased mTOR levels (Figure 4i,j), further indicating that the S1P‐PS‐MMV@SPD promoted the Treg polarization via autophagy. We further evaluated the influence of EMNV‐induced M2 macrophages on T cell differentiation. The percentage of CD3^+^CD4^+^Foxp3^+^ Tregs increased slightly when co‐cultured with M0 macrophages and MMV‐treated M0 macrophages compared to that of the control group (Figure S12). Both S1P‐PS‐MMV‐ and S1P‐PS‐MMV@SPD‐treated M0 macrophages showed a significant increase in the percentage of CD3^+^CD4^+^Foxp3^+^ Tregs. Notably, S1P‐PS‐MMV@SPD treated M0 macrophages exhibited the most substantial promoting of Treg differentiation. These results were consistent with the data presented in Figure 2d, where S1P‐PS‐MMV@SPD‐treated M0 macrophages demonstrated the highest percentage of F4/80^+^CD206^+^ M2 macrophages and secreted the highest amount of anti‐inflammatory cytokines compared to the other treatment groups. This results suggested that EMNVs also can indirectly influence Treg differentiation by inducing M2 macrophages, demonstrating its potential in regulating immune responses. EMNVs may play a crucial role in both innate and adaptive immune cells regulation. The S1P‐PS‐MMV without SPD loading primarily had an anti‐inflammatory effect on macrophages, while S1P‐PS‐MMV@SPD not only possessed the ability to regulate macrophages, whereas the S1P‐PS‐MMV@SPD not only promoted macrophage differentiation towards an anti‐inflammatory phenotype, but also regulated T cell levels by directly or indirectly inducing their transformation.

### 2.4 Tissue Targeting of EMNV in vivo

**Figure 5:**
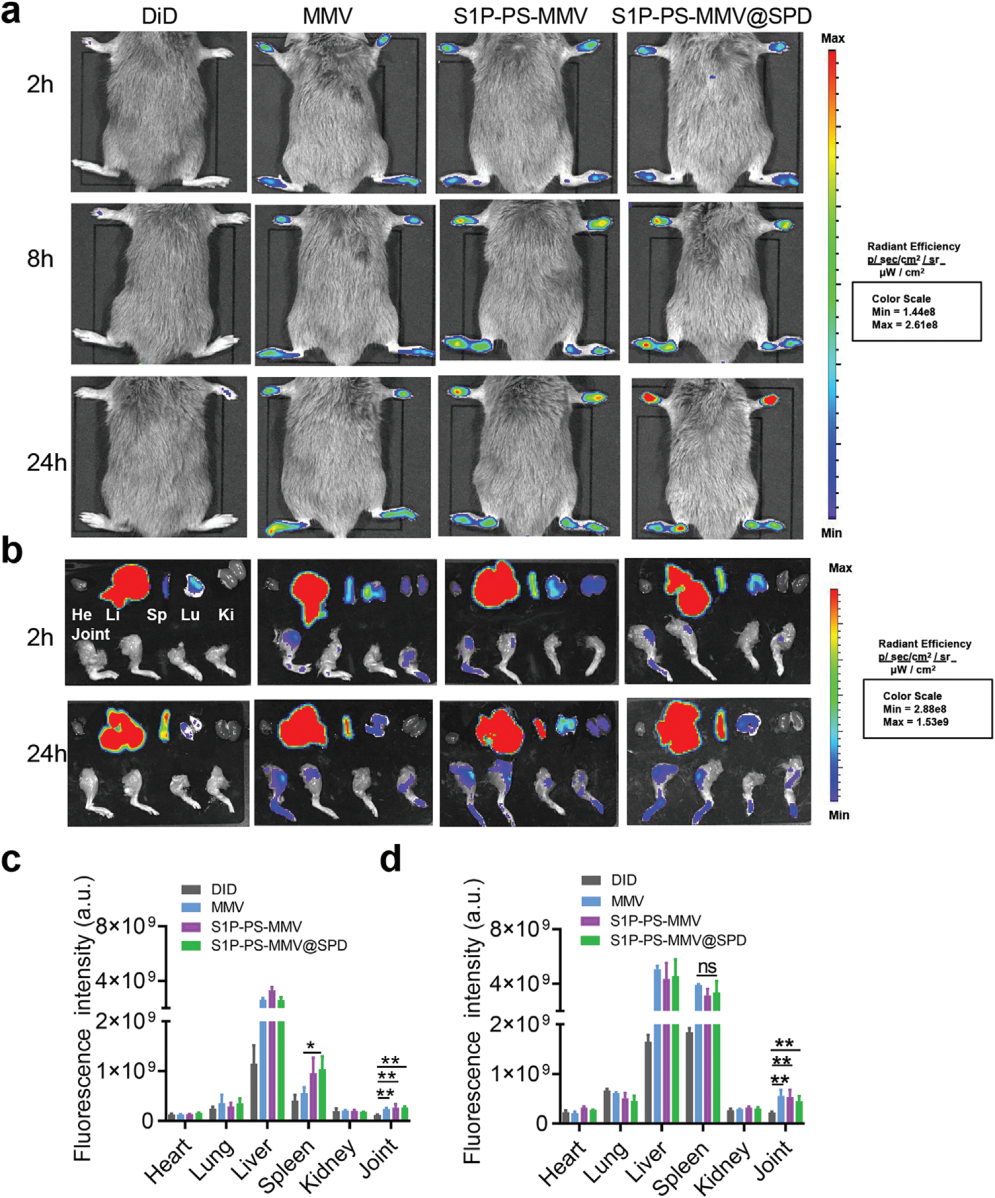
Inflammatory joint‐ and spleen‐targeting capability of EMNVs in vivo. a) Representative fluorescence images of vesicles accumulation in morbid paws at different time points after i.v. injections. b) Ex vivo tissue distribution of vesicles at different time points. He: heart; Li: liver; Sp: spleen; Lu: lung; Ki: kidney; Joint: joint. Vesicles were labeled using DiD fluorescence. Quantitative analysis of fluorescent signals of vesicles in main organs and paws at 2 h c) and 24 h d). Data are expressed as mean ± SD (n = 3). ^*^ *p* < 0.05, ^**^ *p* < 0.01.

The biodistribution of EMNVs was assessed in the CIA mouse model. The mice received an intravenous injection of free DiD solution, DiD‐labeled MMV, S1P‐PS‐MMV or S1P‐PS‐MMV@SPD and were then subjected to in vivo imaging. Fluorescence signals were observed in the morbid paws of the MMV, S1P‐PS‐MMV, and S1P‐PS‐MMV@SPD treatment groups, however, no fluorescence signal was observed in the free DiD treatment groupAs shown in (**Figure** **5**a). Fluorescence signal intensity increased in the inflamed paw area over time. In the MMV, S1P‐PS‐MMV, and S1P‐PS‐MMV@SPD treament groups, the signals were retained at the RA‐inflamed site for >24 h. This was attributed to the adhesion proteins (CD44 and Mac‐1) on the surface of macrophage membranes that could effectively target and bind to inflamed endothelial cells.^[^ ^46^ ^]^ Ex vivo imaging of the main organs after 2 h and 24 h showed that the fluorescence signals in the MMV, S1P‐PS‐MMV, and S1P‐PS‐MMV@SPD treatment groups were mainly distributed in the liver, spleen, and inflamed paws. The S1P‐PS‐MMV or S1P‐PS‐MMV@SPD treatment groups exhibited stronger signals in the spleen and inflamed joints (Figure 5b). Compared to 2 h, the fluorescence intensity in the spleen and inflamed paw increased after 24 h, whereas there was no change in the liver (Figure 5c,d). Because the spleen is the primary blood filter, the intravenous infusion of apoptotic cells is expected to be mainly eliminated in the spleen.^[^ ^34^ ^]^ Furthermore, we investigated the biodistribution of EMNVs in normal mice and different immune cells (dendritic cells, macrophages, neutrophils and T cells) in the spleen using flow cytometry. EMNVs accumulated in the spleen and were recognized and engulfed by immune cells in the spleen, rather than in the joints of normal mice (Figures S13 and S14), indicating the potential of EMNVs for modulating immune cell differentiation and targeting inflamed tissue. The accumulation of EMNVs at the inflammatory site and in the spleen suggested that the S1P‐PS‐MMV@SPD may regulate T cell differentiation in the spleen.

### 2.5 Therapeutic Effect of EMNV in RA Mice

**Figure 6:**
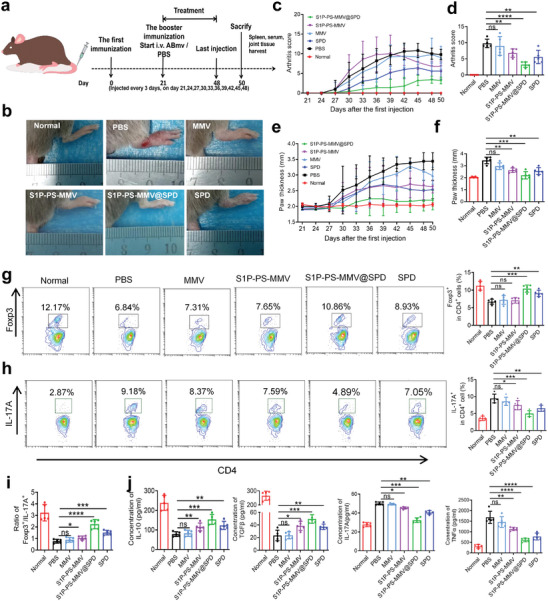
The therapeutic effect of EMNV in RA model. a) Timeline of EMNV treatment experiment in CIA model. b) Representative macroscopic images of hind paw on day 50 after different treatments. c) Change in arthritis scores in mice after different treatments and d) the average of arthritis scores on day 50. e) Change in hind paw thickness after different treatments and f) the average of paw thickness on day 50. g) Representative flow cytometric plots indicating the percentage of CD3^+^CD4^+^Foxp3^+^ Tregs and h) CD3^+^CD4^+^IL‐17A^+^ Th17 cells in the spleen of CIA mice after different treatments on day 50. i) Ratio of Treg/Th17 cells in spleen of CIA mice after different treatments on day 50. j) Serum levels of TNF‐α, IL‐17, IL‐10, and TGF‐β in CIA mice after different treatments on day 50. Healthy mice served as the normal group. Data are expressed as mean ± SD (n = 5). ^*^p < 0.05, ^**^p < 0.01, ^***^p < 0.005, ^****^p < 0.0001, ns = no significance.

To evaluate the potential impact of EMNVs on RA, CIA model mice were treated every 3 d, according to the schedule in **Figure** **6**a. Paw swelling, often considered as an indicator of arthritis, was recorded over time. Compared to the PBS treatment, MMV, S1P‐MMV, and PS‐MMV treatments did not alleviate paw swelling. In contrast, S1P‐PS‐MMV, S1P‐PS‐MMV@SPD, and free SPD treaments reduced paw swelling and redness. Notably, the S1P‐PS‐MMV@SPD treatment was associated with the lowest swelling (Figure 6b and S15a). The S1P‐PS‐MMV@SPD treatment resulted in the lowest paw thickness and arthritis score (Figure 6c–f and S15b–e), similar to those in the normal group, confirming the advantages of the S1P‐PS‐MMV@SPD in targeting inflammation and regulating T cell differentiation. The percentages of Treg and Th17 cells in the spleen were evaluated by flow cytometry. Both S1P‐PS‐MMV@SPD and SPD treatments increased the proportion of Tregs (Figure 6g,h and S15f,g), whereas the proportion of Th17 cells was reduced, and lower in the S1P‐PS‐MMV@SPD treatment group than that in the SPD treatment group. However, there was no clearchange in the percentage of Treg and Th17 cells after MMV treatment compared to PBS treatment. Meanwhile, compared to the PBS treatment, the ratio of Treg/Th17 cells was highest following the S1P‐PS‐MMV@SPD treatment, followed by the SPD treatment, whereas the S1P‐PS‐MMV treatment showed a slight increase (Figure 6i and S15h). The percentage of CD3^+^CD4^+^Foxp3^+^ Tregs was ∼9% in the lymph nodes across all treatment groups (MMV, S1P‐PS‐MMV, S1P‐PS‐MMV@SPD, and SPD), without any significant difference compared with that in the PBS treatment group (Figure S16). These findings indicated that EMNVs did not alter CD3^+^CD4^+^Foxp3^+^ Treg levels in the lymph nodes, however, further studies are required to elucidate the underlying mechanisms. We detected the serum levels of inflammation‐ (TNF‐α and IL‐17) and anti‐inflammation‐related cytokines (IL‐10 and TGF‐β) in RA mice using enzyme‐linked immunosorbent assay (ELISA), to assess the potential systemic immune response. Compared to the PBS treatment, the administration of MMV, S1P‐MMV, and PS‐MMV showed negligible changes in the serum levels of inflammatory and anti‐inflammatory factors (Figure 6j and S15i), suggesting that MMV modifications with either S1P or PS alone resulted in a weaker anti‐inflammatory effect in vivo. However, compared to the S1P‐PS‐MMV treatment, the S1P‐PS‐MMV@SPD treatment resulted in higher levels of IL‐10 and TGF‐β, along with lower levels of TNF‐α and IL‐17, demonstrating that SPD loading further enhanced the therapeutic efficacy of the EMNV. These results suggested that the S1P‐PS‐MMV@SPD modulated the Treg/Th17 cell balance and induced systemic immunosuppression in RA.

**Figure 7:**
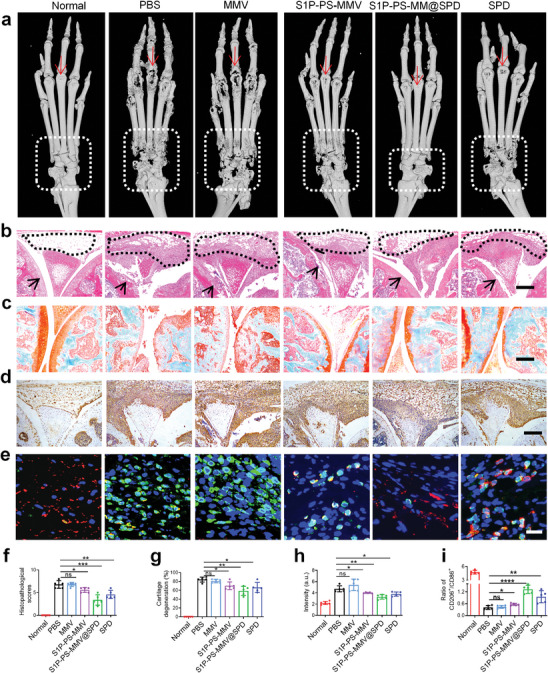
Effect of EMNV on joint injury and inflammatory response in CIA model. a) Representative micro‐CT images of arthritic paws in different treatment, The arrows indicated the symptoms or progression trend of finger joint injury, the dashed boxes represented the range or affected area of ankle joint injury. b) H&E images, c) Safranin‐O/fast green staining, and d) immunohistochemical staining images of histological sections in joints after different treatments. Scale bar = 200 µm. The scale bar in the far‐right image applies to all the images. Aarrows indicate cartilage degradation, triangles represent synovial hyperplasia. e) Immunostaining images of CD86 and CD206 (green: CD86; red: CD206; blue: DAPI; scale bar = 20 µm). These images are the close‐up of the area within the white dashed boxes in the Figure S17. f) Histopathological evaluation of synovial inflammation and g) quantified cartilage degeneration levels were obtained from the images in (b) and (c). h) Mean intensity of TNF‐α. i) Quantitative analysis of relative of CD206^+^/CD68^+^ macrophage ratios. Data are expressed as mean ± SD (n = 5). ^*^ *p* < 0.05, ^**^ *p* < 0.01, ^***^ *p* < 0.005, ^****^ *p* < 0.0001, ns = no significance.

To further investigate the therapeutic effects of EMNVs against RA, we evaluated cartilage destruction and synovial inflammation in joint tissues using micro‐computed tomography (micro‐CT), hematoxylin and eosin (H&E) staining, Safranin‐O staining, and immunostaining, respectively. The PBS and MMV treatments resulted in obvious bone corrosion in the ankle and toe joints (**Figure** **7**a). In contrast, the S1P‐PS‐MMV@SPD treatment significantly mitigated the bone erosion, likely due to the increased accumulation of SPD and the anti‐inflammatory effect of PS‐modified vesicles. Compared with PBS and MMV treatments, SPD and S1P‐PS‐MMV treatments resulted alleviated synovitis and cartilage destruction, while the S1P‐PS‐MMV@SPD treatment displayed the mildest synovial hyperplasia and smooth and intact of cartilage structure (Figure 7b,f and S17). Safranin‐O/fast green staining showed that the PBS and MMV treatments resulted in the most severe loss of cartilage proteoglycans. The S1P‐PS‐MMV@SPD treatment efficiently decreased the loss of cartilage proteoglycan by 58.68 ± 10.16%, notably outperforming SPD by 66.34 ± 11.39% and S1P‐PS‐MMV by 68.3 ± 8.62% (Figure 7c,g). These results are consistent with the those of the micro‐CT analysis. Compared with the normal group, the expression of TNF‐α was significantly elevated in the PBS treatment group (Figure 7d,h), reflecting the involvement of these cytokines in the pathogenesis of RA. There were no significant differences in the expression levels of TNF‐α between the SPD and S1P‐PS‐MMV treatments groups. However, the S1P‐PS‐MMV@SPD treatment showed significantly reduced TNF‐α expression, consistent with the micro‐CT, H&E and safranin‐O/fast green staining results. The synovial macrophage phenotype was evaluated by immunofluorescence staining with anti‐CD206 (M2 marker) and anti‐CD86 (M1 marker). As shown in Figure 7e,i and wide‐field view of joint tissue sections (Figure S18), the S1P‐PS‐MMV@SPD treatment clearly alterated the phenotype of synovial macrophages to reduce the inflammation in joint tissues. Finally, we assessed the long‐term biocompatibility and hepatotoxicity of EMNV in vivo. The H&E staining images revealed no observable histologic damage in any other organs of the mice treated with EMNVs (Figure S19). Meanwhile, the levels of liver toxicity‐related indicators in the serum, specifically alanine aminotransferase (ALT), alkaline phosphatase (ALP), and aspartate aminotransferase (AST), were all within normal ranges (Figure S20). These findings indicated the absence of significant liver damage, suggesting negligible systemic toxicity over 4 weeks of the S1P‐PS‐MMV@SPD treatment. Collectively, these results revealed that the S1P‐PS‐MMV@SPD modulated articular inflammation, and regulated systemic immunity by maintaining healthy M1/M2 macrophage and Treg/Th17 cell ratios, presumably facilitating the therapeutic effect in RA.

## 3 Conclusions

We successfully designed and prepared the S1P‐PS‐MMV@SPD, which accurately mimics the functionalities of efferocytosis by integrating a “find me” signal (S1P), “eat me” signal (PS), and metabolites representative of apoptotic cells (SPD) within nanovesicles. We demonstrated the effective induction of migration and recognition of macrophages and T‐lymphocytes by the S1P‐PS‐MMV@SPD. Upon apoptotic cell engulfment, the S1P‐PS‐MMV@SPD exerted anti‐inflammatory and immunosuppressive effects through multiple mechanisms, including the promotion of M2 macrophage polarization, enhancement of Treg differentiation, and inhibition of Th17 cell levels. In vivo experiments confirmed that the S1P‐PS‐MMV@SPD accumulated simultaneously at the arthritis site and in the spleen, effectively suppressing the progression of RA by regulating the imbalance between M1/M2 macrophages in the synovium and Th17/Treg cells in the spleen. However, further investigation was warranted to provide a comprehensive understanding of the precise molecular mechanisms and signaling pathways underlying T cell regulation by the S1P‐PS‐MMV@SPD. Additionally, long‐term monitoring and experim entation are needed to assess the potential of the S1P‐PS‐MMV@SPD in the treatment of RA, which should preferably consider various animal models. In conclusion, the novel nanovesicle‐based efferocytosis‐mimicking S1P‐PS‐MMV@SPD exhibited a dual function of regulating joint inflammation and suppressing peripheral immunity, demonstrating great potential for application in the clinical treatment of RA.

## 4 Experimental Section

### Animals

C57BL/6 mice (male, 7–8 weeks old) and DBA/1 mice (male, 7–8 weeks old) were purchased from Hangzhou Medical College and Beijing Vital River Experimental Animal Technology, respectively. All animals were handled in compliance with the guidelines in the Guidance Suggestions for the Care and Use of Laboratory Animals. This study was approved by the Experimental Animal Ethics Committee of Wenzhou Institute, University of Chinese Academy of Sciences (approval nos.WIUCAS21122202 and WIUCAS22032305).

### Macrophage membranes isolation

RAW264.7 cells were purchased from the Sciences Shanghai Institites for Biological Sciences Cell Resource Center (Chinese Academy of Sciences). The cells were cultured in high glucose Dulbecco's modified Eagle's medium (DMEM, Gibco, USA) supplemented with 10% fetal bovine serum (FBS), penicillin (100 U mL^−1^) and streptomycin (100 mg mL^−1^) at 37 °C in 5% CO_2_ atmosphere. Macrophage membranes were isolated as previously described.^[^ ^58^ ^]^ Briefly, the cells were washed three times using PBS, and then dispersed in homogenization buffer containing 30 mM Tris‐HCl (pH = 7.5), 75 mM sucrose, 0.5 mM EDTA, 225 mM mannitol and a protease and phosphatase inhibitor cocktail (Shanghai Epizyme Biomedical Technology Co., Ltd). After cooling for 15 min on ice, the solution was sonicated using a homogenizer for seven passes at 30% power. The supernatant was gathered and centrifuged at 21 000 ×g for 30 min. The obtained pellet was used as the plasma membranes in subsequen experiments. The protein content of the obtained membranes was quantified using a bicinchoninic acid assay (Biosharp, China).

### Preparation and characterization of EMNVs

The collecting macrophage membranes were sonicated using a homogenizer (VCX 130 PB, Sonics, USA) for 3 seconds, and then extruded twice through a 5 µm polycarbonate porous membrane to obtain macrophage membranes‐derived vesicles (MMVs). We dissolved 1,2‐dioleoyl‐sn‐glycero‐3‐phospho‐L‐serine (PS, Aladdin) in a mixture of chloroform and methanol (volume ratio = 4:1) solution, and the solvent was completely removed by rotary evaporation. The obtained PS film was diluted in sterile deionized water to 5 mg mL^−1^ and stored at –20 °C in the dark. The EMNV was prepared by inserting PS and S1P onto the surface and loading SPD inside the MMV using the co‐extrusion method. Briefly, the mixture of MMV, S1P, and PS (mass ratio of 80:8) was extruded through a 5 µm polycarbonate porous membrane to prepare the S1P‐PS‐MMV. The S1P‐PS‐MMV@SPD was obtained by co‐extrusion of the S1P‐PS‐MMV with SPD through a 5 µm polycarbonate porous membrane.

The morphology and structure of the EMNV were confirmed using TEM. The size and zeta (ζ)‐potential of the EMNV were analyzed by dynamic light scattering (DLS, ZEN 3600 Zetasizer, Malvern). Serum stability was examined by mixing 1 mg mL^−1^ of EMNVs in PBS with 10% FBS for 3 d. The insertion of PS in EMNVs was detected through flow cytometry (CytoFLEX, USA) analysis using a fluorescein‐labeled Annexin V biomarker (eBioscience, USA). To detect the membrane proteins in the macrophage membranes, the S1P‐PS‐MMV and S1P‐PS‐MMV@SPD were extracted using a membrane protein extraction kit (Beyotime, Shanghai, China) following the manufacturer's protocols. The protein composition of the EMNV was determined by SDS‐PAGE. The specific surface markers (CD44 and Mac‐1) on the macrophage membranes, MMV, S1P‐PS‐MMV, and S1P‐PS‐MMV@SPD were investigated by western blot analysis. To evaluate the release performance of S1P and SPD under physiological and acidic conditions, EMNVs were dispersed in a 2 mL EP tube containing 1 mL PBS (pH 5.0 or pH 7.4), and then incubated at 37 °С in a thermostat water bath with a shaking speed of 100 × rpm. At specific time intervals, the sample were centrifuged at 21 000 ×g for 5 min to obtain the supernatant and equal amounts of fresh PBS were added. The concentrations of S1P and SPD were measured as previously described.^[^ ^59^, ^60^ ^]^ The corresponding procedures are described in the SI Experimental Section.

### Isolation and culturation of mouse primary immune cells

Bone marrow‐derived monocytes (BMDMs) were generated from bone marrow cells isolated from the tibias and femurs of C57BL/6 mice and cultured in DMEM supplemented with 10% FBS, 15% L929‐conditioned medium and pen/strep for 7 d at 37 °C in 5% CO_2_. Fresh complete medium was changed on day 4, and BMDMs cultured for 7 d were used as M0 macrophages.^[^ ^61^ ^]^ For M1 macrophage polarization, M0 macrophages were incubated with recombinant mouse INF‐γ (20 ng mL^−1^) and LPS (100 ng mL^−1^) for 24 h. T lymphocytes were collected from the spleen by negative selection using a mouse T cell isolation kit (Precision BioMedicals Co., Ltd) according to the manufacturer's instructions. The purity of the isolated T cells was determined using flow cytometry measurement. Approximately 2 × 10^6^ isolated T cells were cultured in 24‐well plates precoated with anti‐CD3 (1 µg mL^−1^) and anti‐CD28 (µg mL^−1^). For Th17 cell differentiation, TGF‐β (1 ng mL^−1^), IL‐6 (10 ng mL^−1^), IL‐1β (10 ng mL^−1^) and IL‐23 (20 ng mL^−1^) were supplemented to the medium.

### Cytotoxicity assay

BMDMs, T cells and L929 cells were seeded separately in a 96‐well plates (3.0 ×10^3^ cells/well) and cultured for 24 h. After reaching approximately 80% confluence, the cells were treated with EMNVs. After co‐culturing for 24 h, the cells were washed three times with PBS, and then fresh medium containing 10% cell counting kit‐8 (CCK‐8) reagent was added for 2 h of incubation in the dark. The optical density (OD) at 450 nm was measured using a microplate reader (ELx800, Bio‐Tek, USA). Cell viability was determined using a CCK‐8 assay (Dojindo, Kumamoto, Japan) according to the manufacturer's instructions.

### Migration assay

A transwell migration assay was performed to test the effect of EMNV on macrophages and T cells recruitment. We resuspended 1×10^5^ cells in medium containing pen/strep and separately seeded on the upper chamber, medium with or without EMNVs was added to the bottom chamber of a 24‐well tissue culture plate with 8 µm pore‐size transwell inserts, and performed incubation for 8 h. The macrophages that migrated into the chamber were fixed and stained. The upper side of the membranes was removed using a cotton swab, and the average number of macrophages on the underside per field was counted using light microscopy. To detect suspended T cells, we counted the number of cells by collecting the medium from the bottom chamber.

### Cellular uptake assay

Macrophage membranes were stained with DiD, (as a cell membrane red‐fluorescent probe), and EMNVs were fabricated using the co‐extrusion method. BMDMs or T cells were seeded in 24‐well tissue culture plates and treated with DiD‐labeled EMNVs at the indicated concentrations. After incubation for 2 h or 24 h, the cells were washed with PBS to remove free EMNVs, and fixed with 4% paraformaldehyde for 30 min. The cells were incubated with DAPI for 5 min and observed using CLSM (Nikon A1). The intensity of DiD was measured using flow cytometry.

### Regulation of EMNVs on macrophages or T cells

M0, M1 macrophages and T cells were cultured separately in the presence of EMNVs for 24 h. We analyzed the indirect regulatory effect of EMNVs on T cells by a co‐culturing EMNV‐treated M0 macrophages with T cells. To determine gene expression, total cellular RNA was extracted using the RNA extraction kit (Sangon Biotech, China), then total RNA was reverse transcribed into cDNA using the reverse transcriptase kit (Takara, Japan), and quantitative real‐time polymerase chain reaction (qRT‐PCR) was performed using a TB‐green PCR Master mix (Takara, Japan). Relative gene expression was calculated using the 2^–△△CT^ method, using glyceraldehyde‐3‐phosphate dehydrogenase (GAPDH) as an internal reference. The primer sequences used for qRT‐PCR analysis are provided in Supplementary Table 2.

Supernatants were collected and the concentration of IL‐10, TGF‑β, IL‐6, TNF‑α, and IL‐17A were determined using an ELISA kit (Peprotech, USA) following the manufacturer's instructions. Flow cytometry was served to detect the phenotypic changes in macrophages and T cells. We measured the expression level of IL‐17A in T cells stimulated with a cell activation cocktail (Biolegend, San Diego, CA, USA) for 5 h before blocking with flow cytometry. The collected cells were blocked in PBS with 1% BSA for 10 min at 4 °C and subjected to surface staining with PE‐conjugated F4/80 (BD Pharmingen, USA), BV21‐conjugated CD86 (BD pharmingen, USA), APC‐conjugated CD3 (Thermo Fisher Scientific, USA), and FITC‐conjugated CD4 antibodies (Thermo Fisher Scientific, USA) for 30 min at 4 °C. For intracellular antibodies, APC‐conjugated CD206, PE‐conjugated Foxp3, and CY7‐conjugated IL‐17A antibodies, the cells were fixed and permeabilized using a fixation/permeabilization kit (BD Pharmingen, USA) before staining. Finally, cells were washed with PBS containing 1% FBS and measured using a flow cytometer.

### Biodistribution of EMNVs in vivo in normal and RA mice

The RA model was established using collagen according to the manufacturer's instructions (Chondrex, USA). Briefly, type II collagen (2 mg mL^−1^, Chondrex) was emulsified with an equal volume of complete Freund's adjuvant (CFA, Chondrex) containing 2 mg mL^−1^ mycobacterium tuberculosis. The emulsion (100 µL) was subcutaneously injected into the tail of DBA/1 mice. On day 21 after the primary injection, DBA mice received a booster injection of type II collagen emulsified in incomplete Freund's adjuvant (IFA, Chondrex). To test the biodistribution of EMNVs in RA mice, DiD‐labeled EMNVs were intravenously injected into mice via the tail vein on day 20 after the booster immunization, and EMNV imaging was performed at a predetermined time using an in vivo imaging system (Caliper, USA). An equal amount of free DiD solution was injected as a control. At 24 h after injection, the mice were sacrificed for major tissue distribution analysis. Further analysis was conducted to examine the uptake of EMNVs by different cells in the spleen of normal mice.

### Therapeutic efficacy of EMNVs in RA mice

We explored the therapeutic efficacy of EMNVs intravenously injected into mice via the tail vein every 3 d, considering the start of treatment as the day of booster immunization. Arthritis scores were recorded over time. The total scores of the four paws of each mouse were evaluated by grading paw swelling as previously described:^[^ ^62^ ^]^ 0 = no erythema or swelling; 1 = mild swelling and one toe inflamed; 2 = mild swelling extending from the ankle to the tarsals; 3 = moderate swelling extending to the entire paw; and 4 = severe swelling extending from the entire paw to the ankle. After 4 weeks, the serum, joint tissues, and spleens were collected. The levels of inflammation (TNF‐α and IL‐17A) and anti‐inflammatory (IL‐10 and TGF‐β) factors in the serum of mice were tested using ELISA. Spleen and lymph nodes cells were extracted after grinding and filtering the tissues through a 70 µm nylon cell strainer (Biosharp, China). The cells were centrifuged at 500 ×g for 5 min, then resuspended in red blood cell lysis buffer and incubated at 4 °C for 10 minutes to remove red blood cells. Finally, the cells were centrifuged again and washed with PBS. Cells isolated from the spleen were analyzed for Treg (CD3^+^CD4^+^Foxp3^+^) and Th17 cell (CD3^+^CD4^+^IL‐17A^+^) levels using flow cytometry.

After 4 weeks of treatment, the hind paws were collected and fixed with 4% PFA for 48 h, and scanned using a micro‐CT imaging system (9 µm, 50 kV, 90 µA, SkyScan 1176, Bruker, Germany). 3D images were reconstructed using CTAn Mimics software (SkyScan). The joints were subjected to histochemical analysis as follows: First, the joints were fixed with 4% PFA for 48 h, decalcified with 10% EDTA for 30 d, embedded in paraffin, and dissected into 5 µm sections, which were stained with H&E, Safranin‐O/fast green solutions to evaluate the synovial and cartilage tissues. Histopathological scoring of synovial inflammation and cartilage destruction was performed in a blinded manner as previously described.^[^ ^18^, ^63^ ^]^ The expression of the inflammatory factor (TNF‐α) in synovial tissues was analyzed via immunohistochemical staining using anti‐TNF‐α (Proteintech, USA). The phenotype of macrophages in the synovial tissues was identified by immunofluorescence staining using anti‐CD206 and anti‐CD68 antibodies (Proteintech), and quantified as the CD206^+^/CD68^+^ ratio. To assess the long‐term biocompatibility and hepatotoxicity of EMNVs in vivo, the major internal organs of treated mice were collected for H&E staining analysis; serum was simultaneously harvested to test liver toxicity‐related indicators (ALT, ALP, and AST).

### Statistical analysis

GraphPad Prism software (Version 8.0) was used for data analysis. Results are displayed as the mean ± standard deviation (SD). Statistical differences between two groups are analyzed using an unpaired two‐tailed Student's *t‐test*. Statistical significance was set at p < 0.05. Statistical significance was assigned as ns (not significant), ^*^ *P* < 0.05, ^**^ *P* < 0.01, ^***^ *P* < 0.001, and ^****^ *P* < 0.0001.

## Conflict of Interest

The authors declare no conflict of interest.

## Supporting information

Supporting Information

## Data Availability

The data that support the findings of this study are available from the corresponding author upon reasonable request.
