# Supplementary material for: Engineering Efferocytosis‐Mimicking Nanovesicles to Regulate Joint Anti‐Inflammation and Peripheral Immunosuppression for Rheumatoid Arthritis Therapy
Source: Adv Sci (Weinh). 2024 May 29;11(28):2404198. doi: 10.1002/advs.202404198 (PMC11267389; doi:10.1002/advs.202404198)
Supplement: Supplementary file 1 — Supporting Information [file ADVS-11-2404198-s001.docx]

Supporting Information

Engineering efferocytosis-mimicking nanovesicles to regulate joint anti-inflammation and peripheral immunosuppression for rheumatoid arthritis therapy

Shanshan Yuan, Yingqian Chai, Jianghua Xu, Youchao Wang, Lihua Jiang, Ning Lu, Hongyi Jiang, Jilong Wang, Xiaoyun Pan,* and Junjie Deng,*

Experimental Section

The measurement of sphingosine 1-phosphate (S1P)

S1P was measured by liquid chromatography with tandem mass spectrometry detection (LC-MS/MS) on Triple QuadTM 4500MD (AB Scies, USA). A C18 chromatographic column (CNW, Athena C18, 2.1 mm × 100 mm, 3 μm) was utilized for chromatographic separation. Samples were separated using a mobile phase A (water with 0.1% formic acid ) and a mobile phase B (methanol with 0.1% formic acid ), and the flow rate was adjusted to 400 μL/min. The total running time of each injection was 5 minutes. The mass spectrometer operated in positive ionization mode (ESI+), and the multiple reaction monitoring (MRM) was established for quantitative analysis. The qualification ion for S1P was optimized as follows: m/z 380.4 →264.4.

The measurement of spermidine (SPD)

Firstly, the sample solution or SPD was diluted to a corresponding concentration with 0.1 mol L^-1^ HCl. One milliliter of saturated NaHCO_3_ was added to 1 mL of sample solution and thoroughly mixed. The pH was then adjusted to 9 with 1 mol L^-1^ of NaOH, followed by the addition of 1 mL of Dns-Cl dissolved in acetone at a concentration of 10 mg/mL. After thoroughly mixing, the solution was incubated at 60 ℃ in a water bath for 45 minutes. Next, 3 mL of ether was added and the mixture was vortexed for 1 minute before being allowed to rest for 2 minutes to allow the layers to separate. The upper organic layer was transferred to a new tube and vacuum-dried. To eliminate the unreacted derivatizing agent, 0.1 mL of 25% ammonia was added after dissolving the residue in 0.9 mL of acetonitrile. Finally, the final product was filtered using a 0.22 mm liquid filter and then subjected to high-performance liquid chromatography (HPLC) analysis.

HPLC with UV-vis spectrometer detector was used to evaluate the final product. A Waters e2695 Separations Module C18 column (4.6 mm, 150 mm, 5 mm) and 2998 PDA Detector were chosen for the separation of the final product of spermidine as the following conditions: the column temperature was set to 20 ℃, the injection volume was 10 μL per injection, the liquid flow rate was 5.0 mL min^-1^ and the UV detection wavelength was 254 nm. The gradient elution procedure consisting of acetonitrile and ultrapure water was carried out as shown in Table 1.


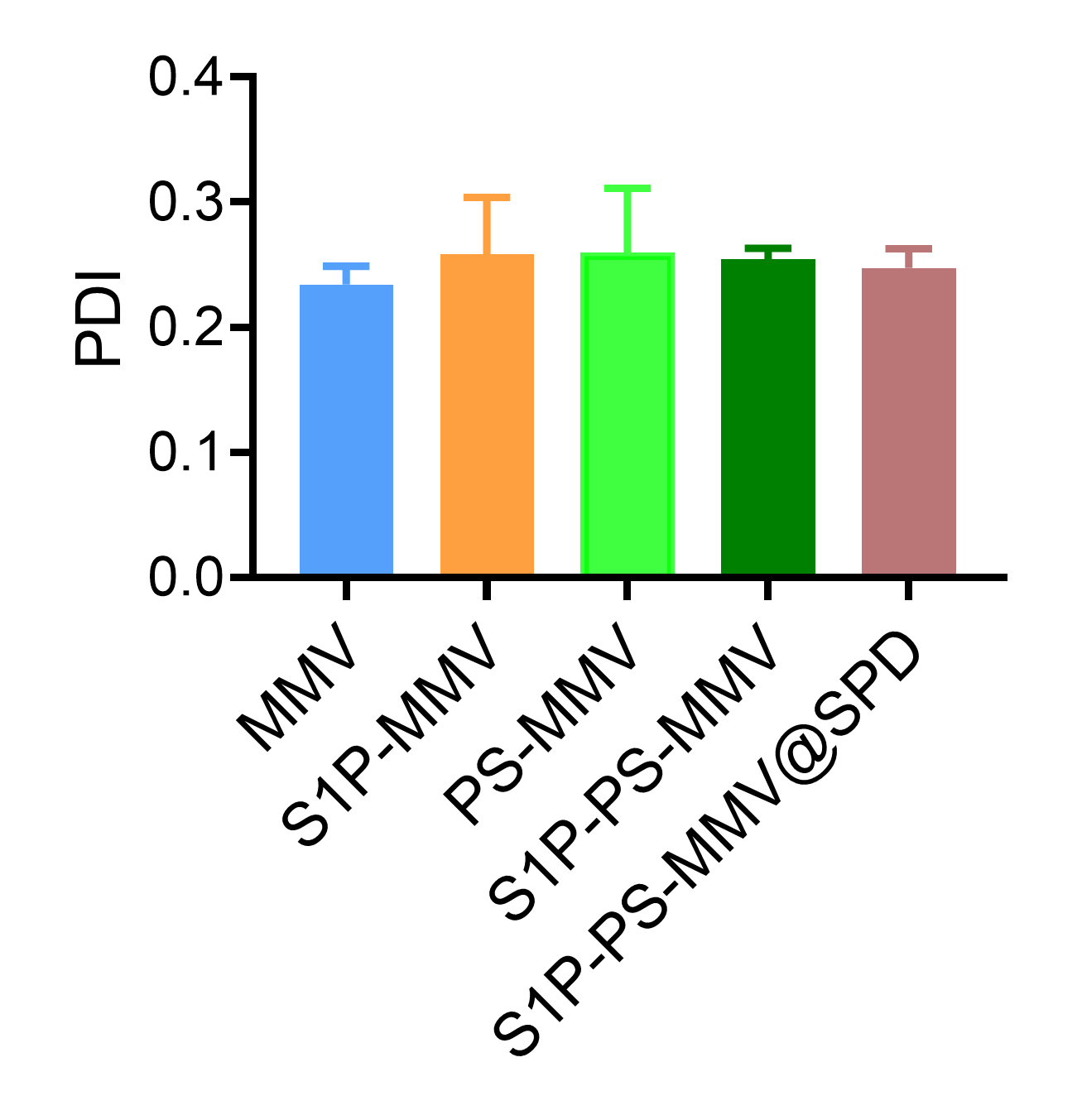


**Figure S1.** The polymer dispersity index (PDI) of vesicles. Data were expressed as mean ± standard deviation (SD) (n=3).


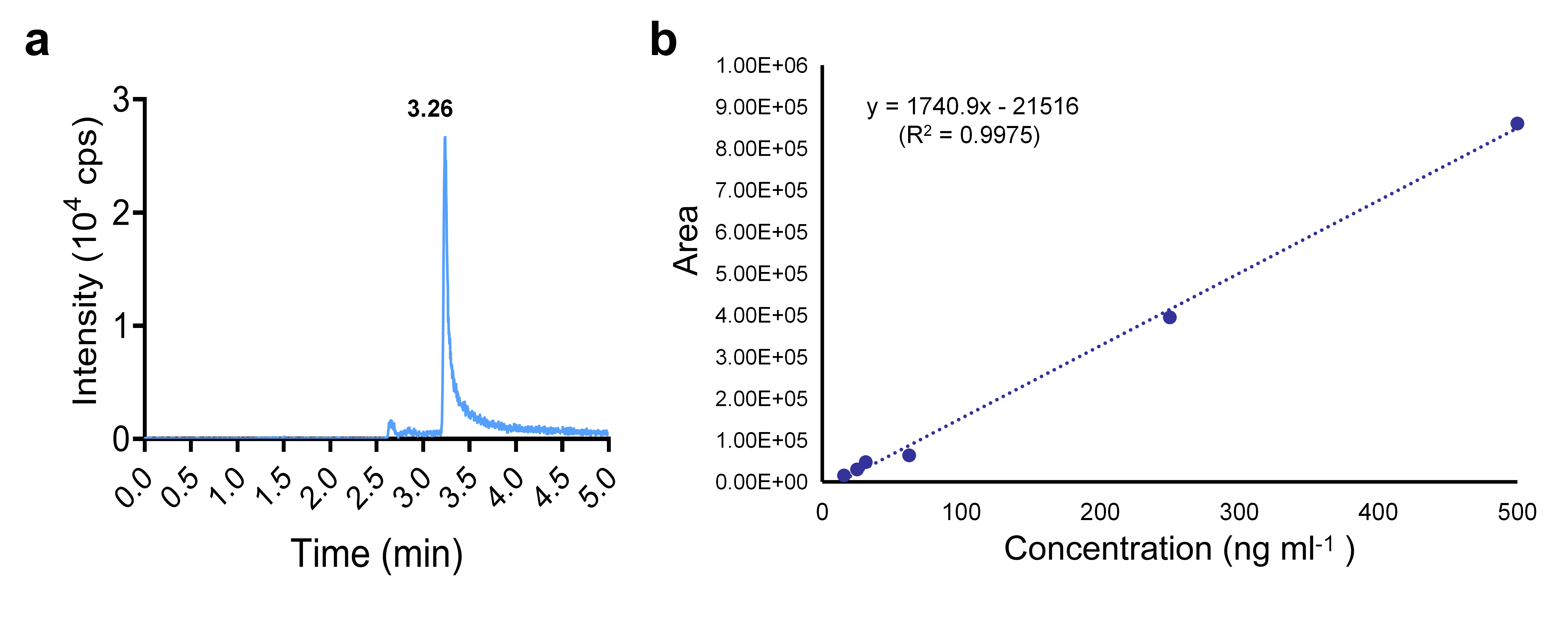


**Figure S2.** The MRM chromatograms for quantification (m/z 380.4/264.5, eluted after 3.26 min) and (b) standard curves of S1P.


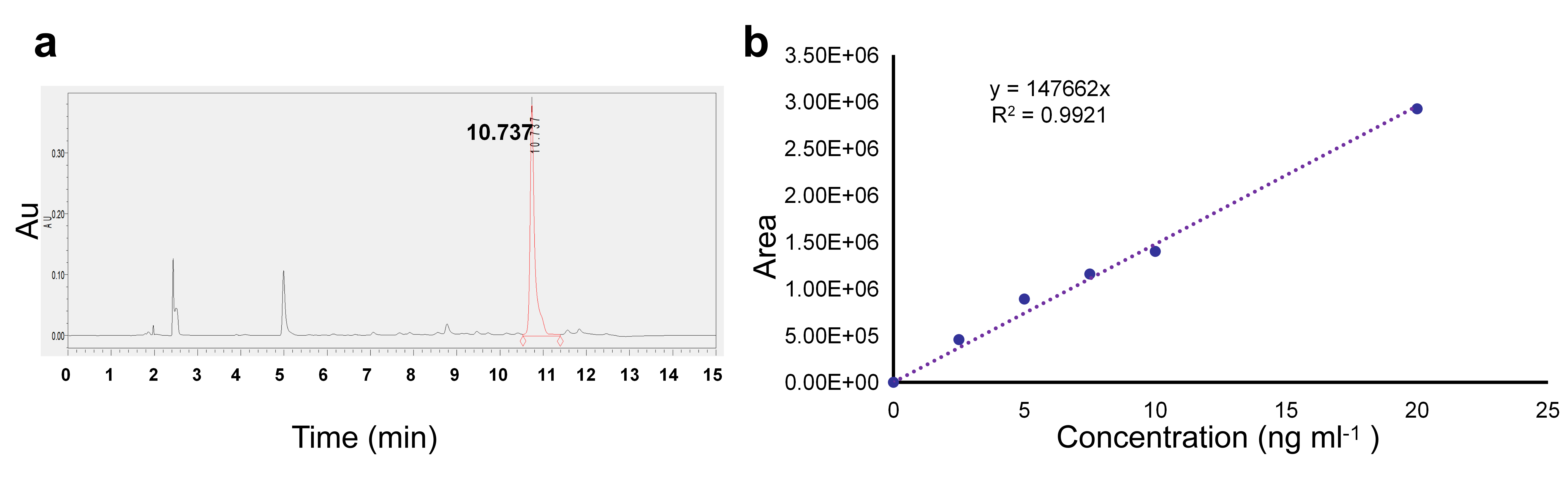


**Figure S3.** The HPLC chromatograms (a) and (b) standard curves for quantification of SPD.


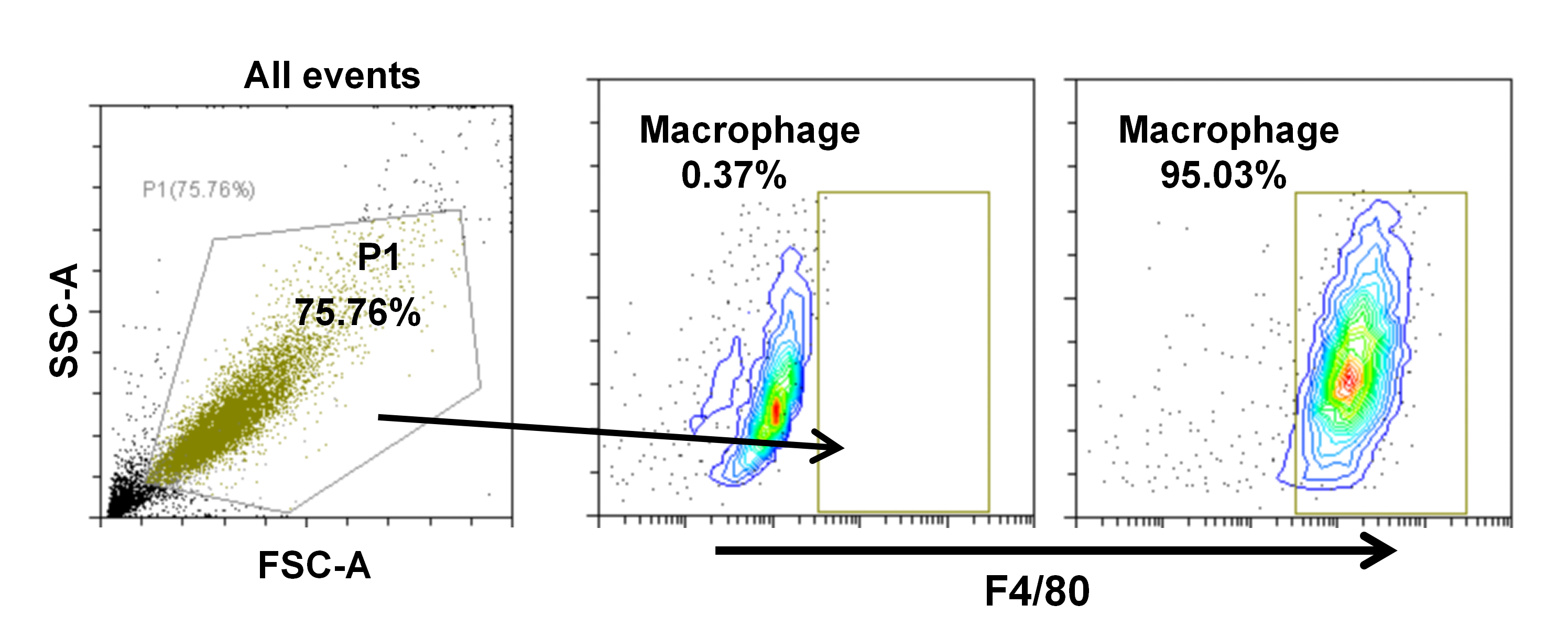


**Figure S4.** Representative flow cytometric chart of mouse bone marrow macrophages (BMDMs) expressing F4/80.


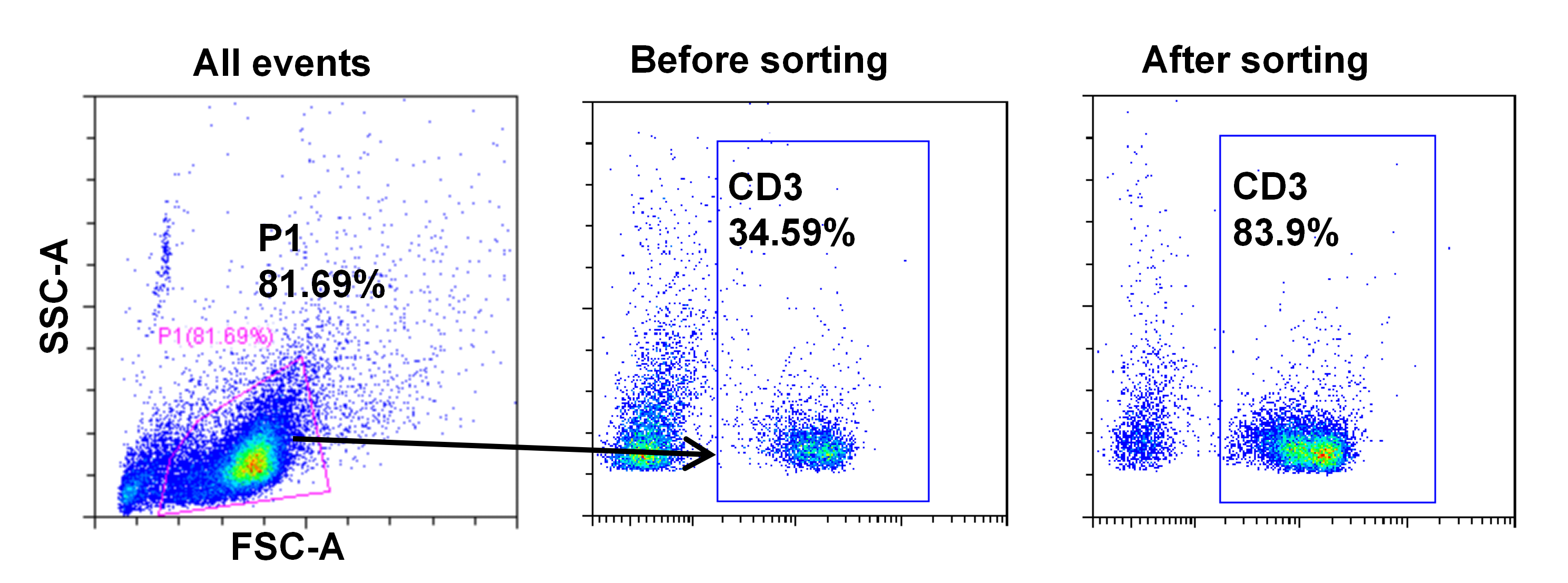


**Figure S5.** Representative flow cytometric plot of CD3 from spleen before and after sorting.


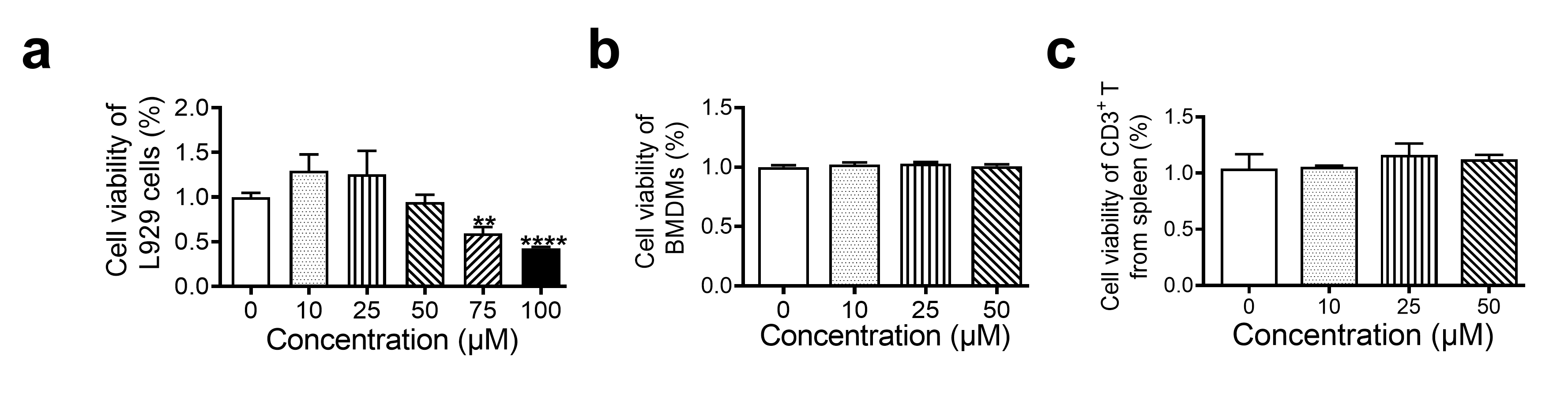


**Figure S6.** The effects of different concentrations of SPD on the cell viability after incubating for 24 h. Data were expressed as mean ± standard deviation(SD) (n=3). *^**^p* < 0.01, *^****^p* < 0.0001.


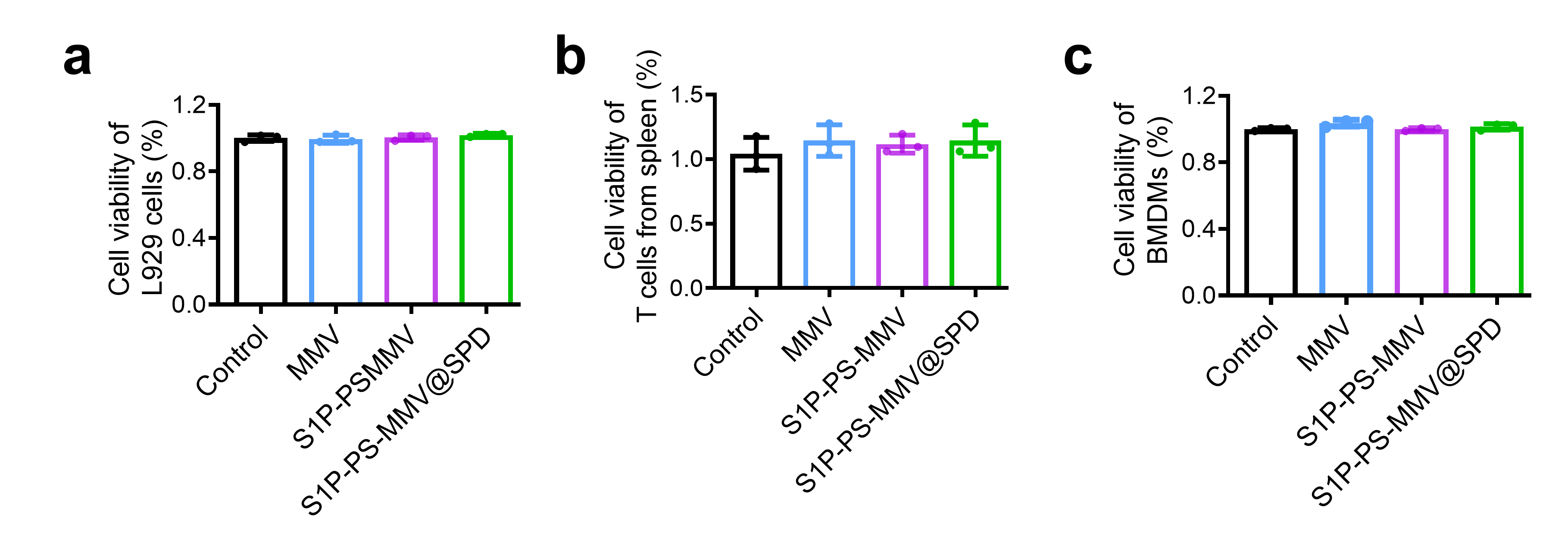


**Figure S7.** The effects of vesicles on cell viability after incubating for 24 h. Data were expressed as mean ± standard deviation(SD) (n=3).


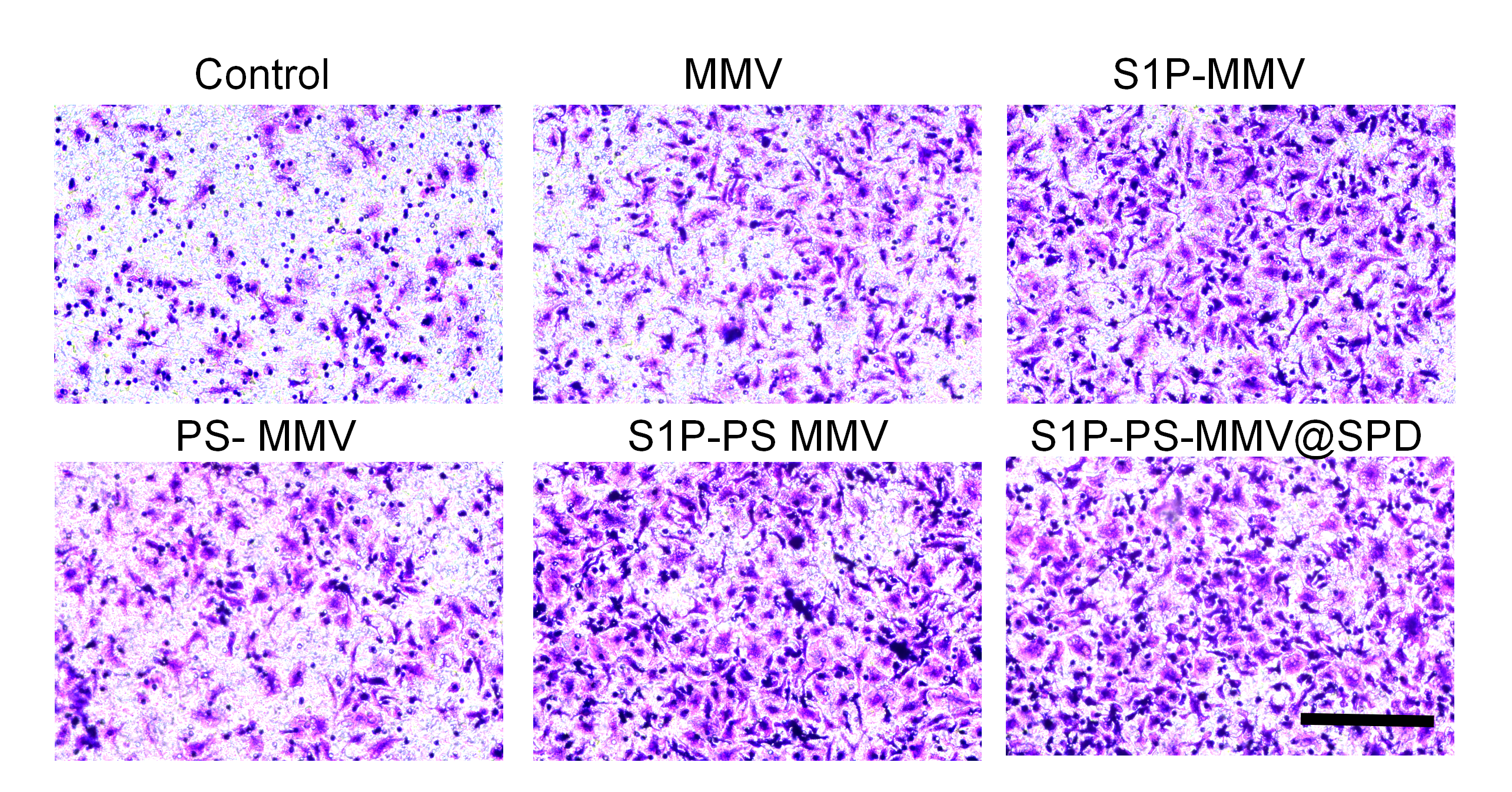


**Figure S8.** Optical micrograph of migration of BMDMs *in vitro* transwell invasion assays. Scale bar = 200 μm.


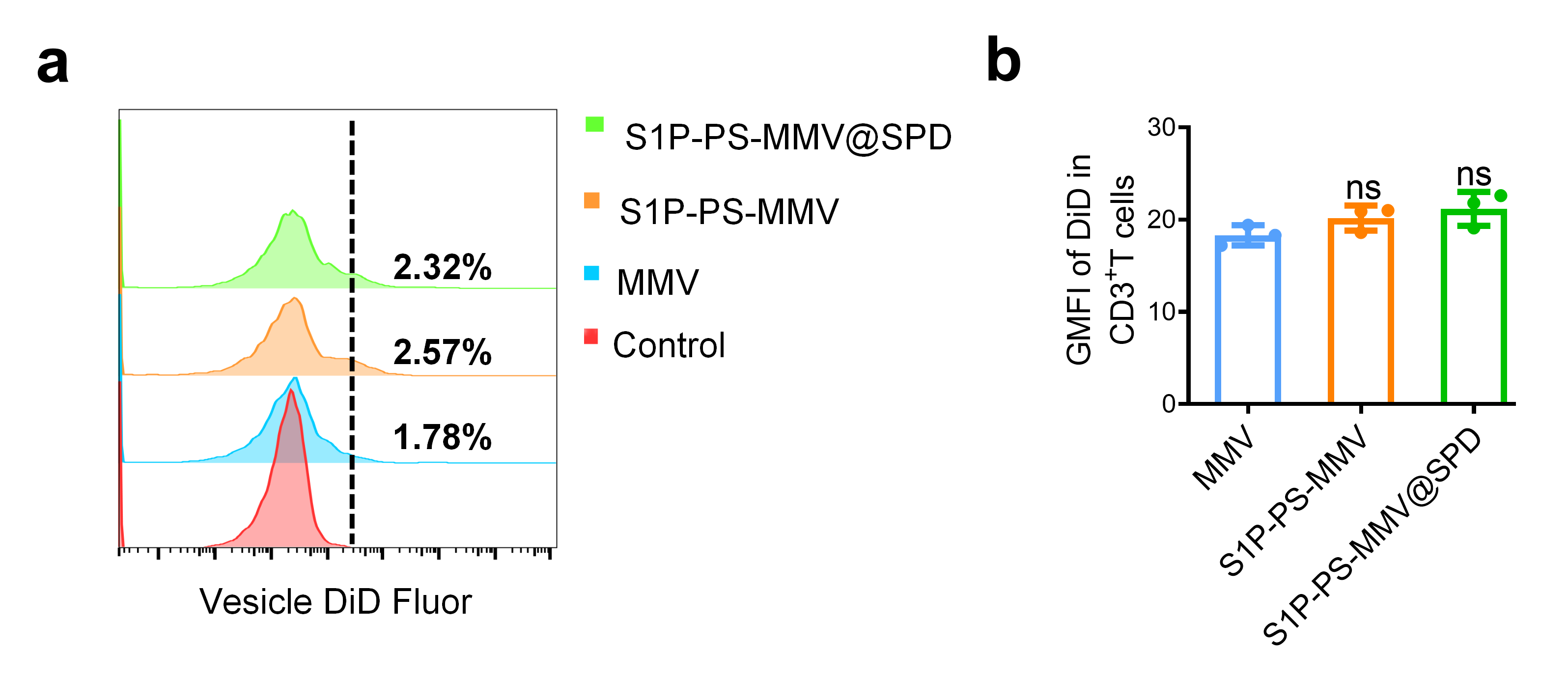


**Figure S9.** Uptake of vesicles by T cells stored from spleen after co-incubation with vesicles for 2 hours *in vitro*. (a) Flow cytometry chart and (b) geometric median fluorescence intensity of DiD signals in T cells after incubation with DiD-labeled vesicle. Data are expressed as mean ± SD (n = 3). ns = no significance.


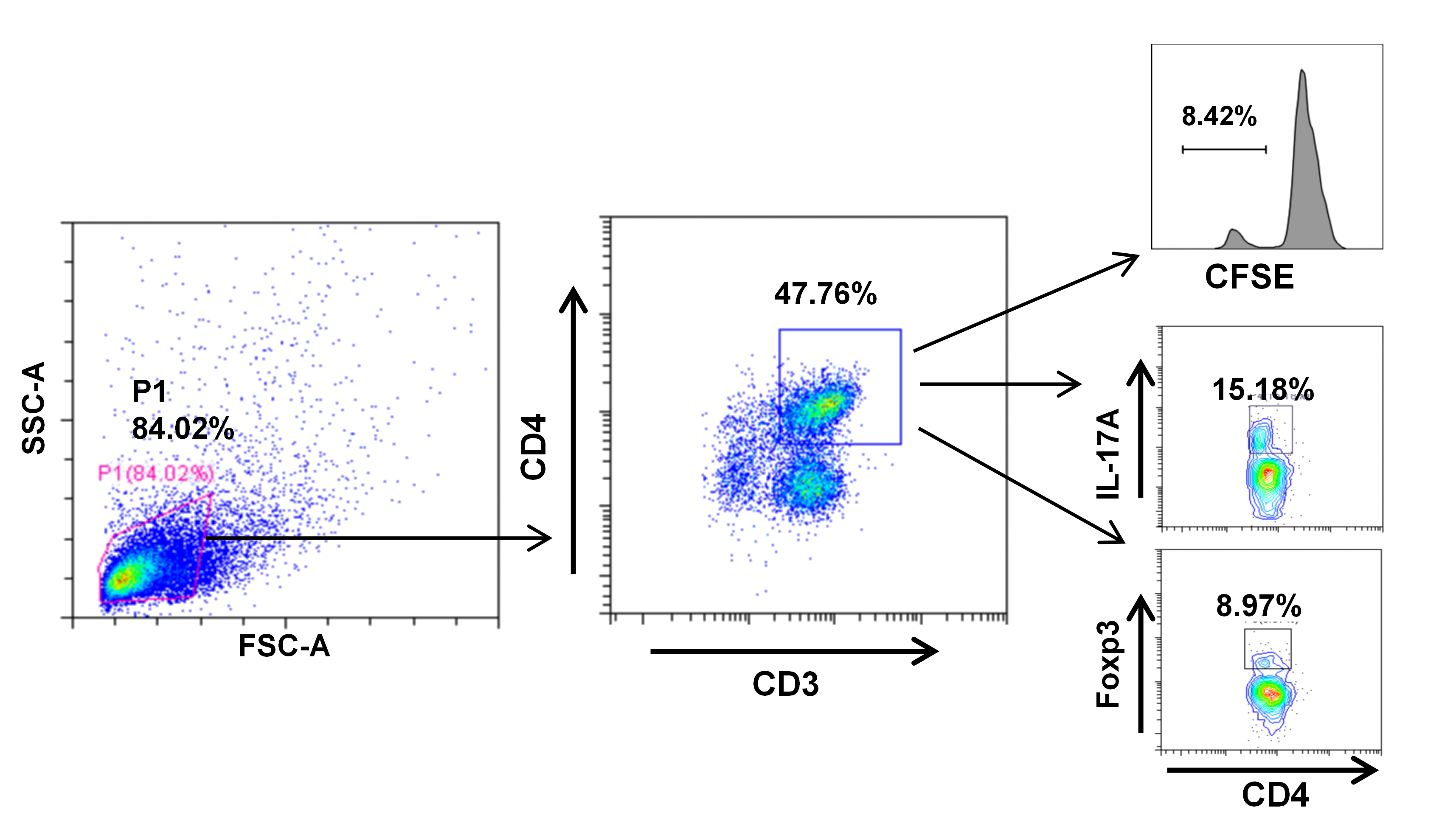


**Figure S10.** The gating strategy diagram of T cell for CFSE, IL-17A, and Foxp3.


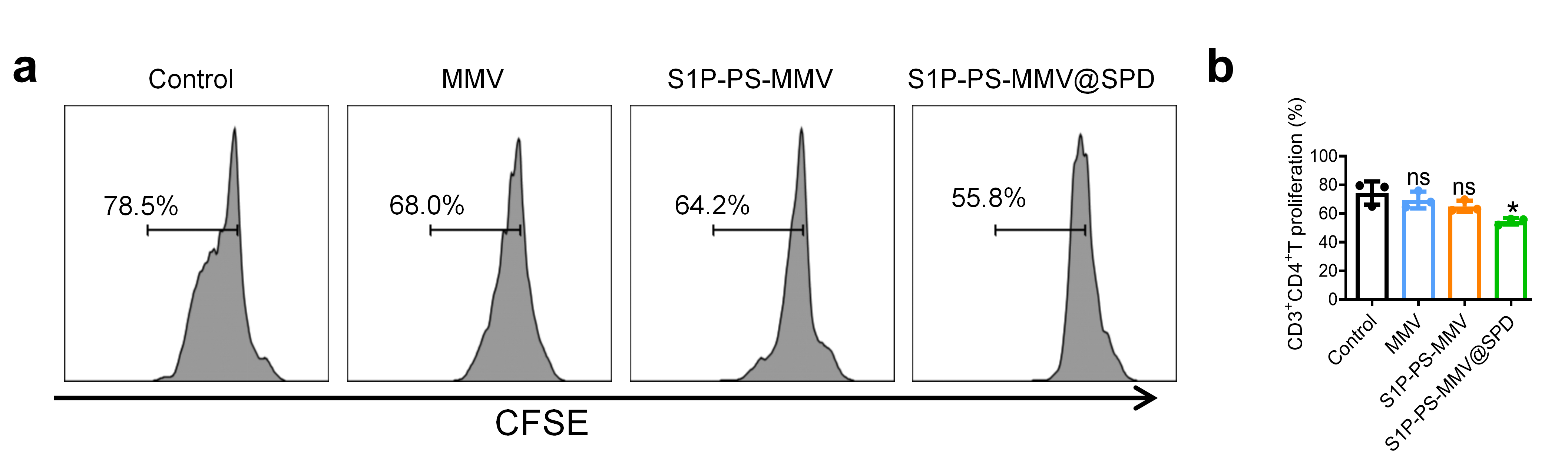


**Figure S11.** The effect of vesicles on the T cell proliferation after incubation for 24 h. (a) Flow cytometry chart and (b) geometric median fluorescence intensity of DiD signals in T cells after incubation with DiD-labeled vesicles. Data are expressed as mean ± SD (n = 3). ^*^*p* < 0.05, ns = no significance.


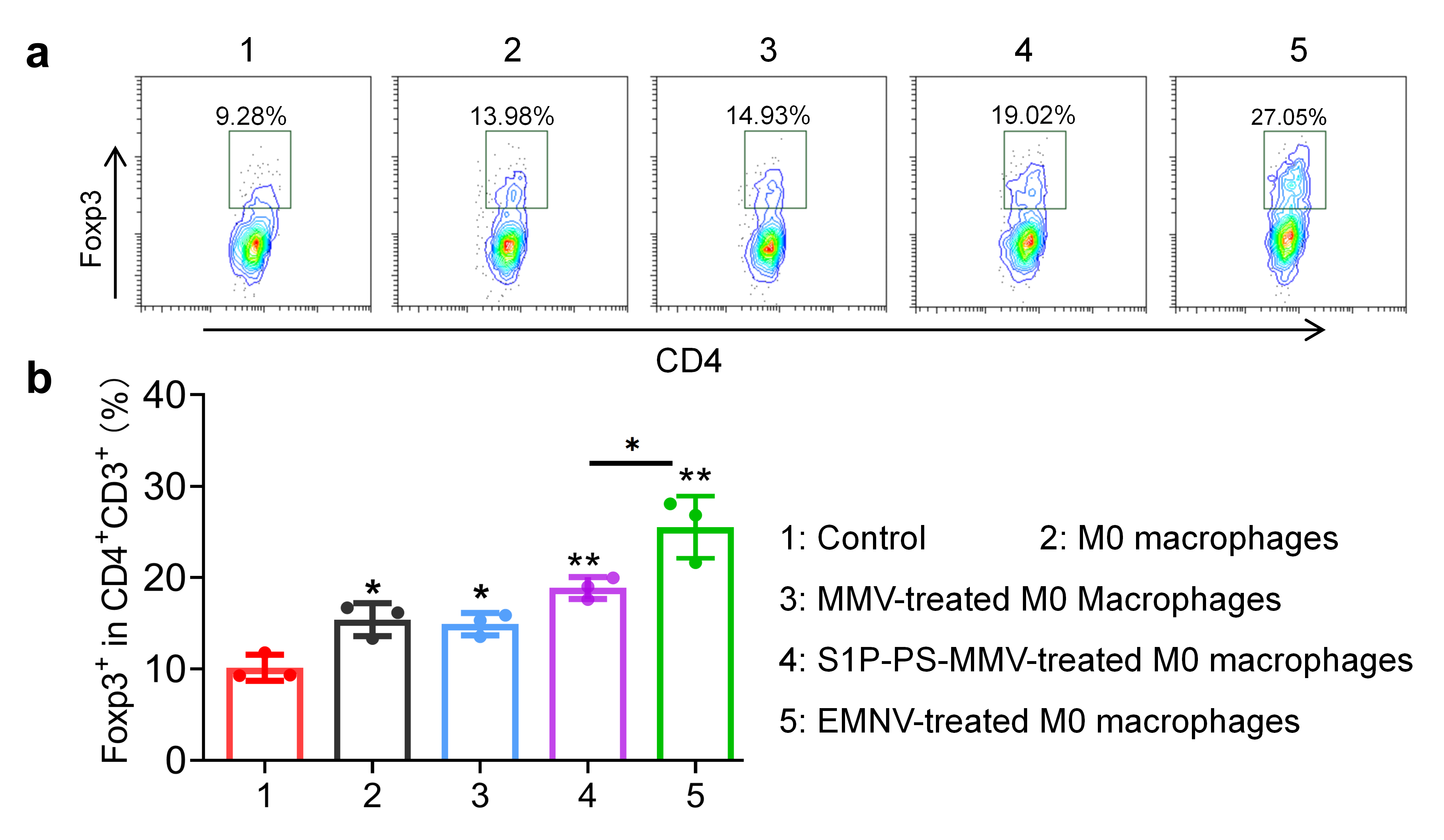


**Figure S12.** The effect of vesicles-treated M0 macrophages on the Treg differentiation after 24 h incubation. (a) Flow cytometry chart and (b) correspondingly quantification analyses of CD3^+^CD4^+^Foxp3^+^ Treg. Data are expressed as mean ± SD (n = 3). ^*^*p* < 0.05, ^**^*p* < 0.01.


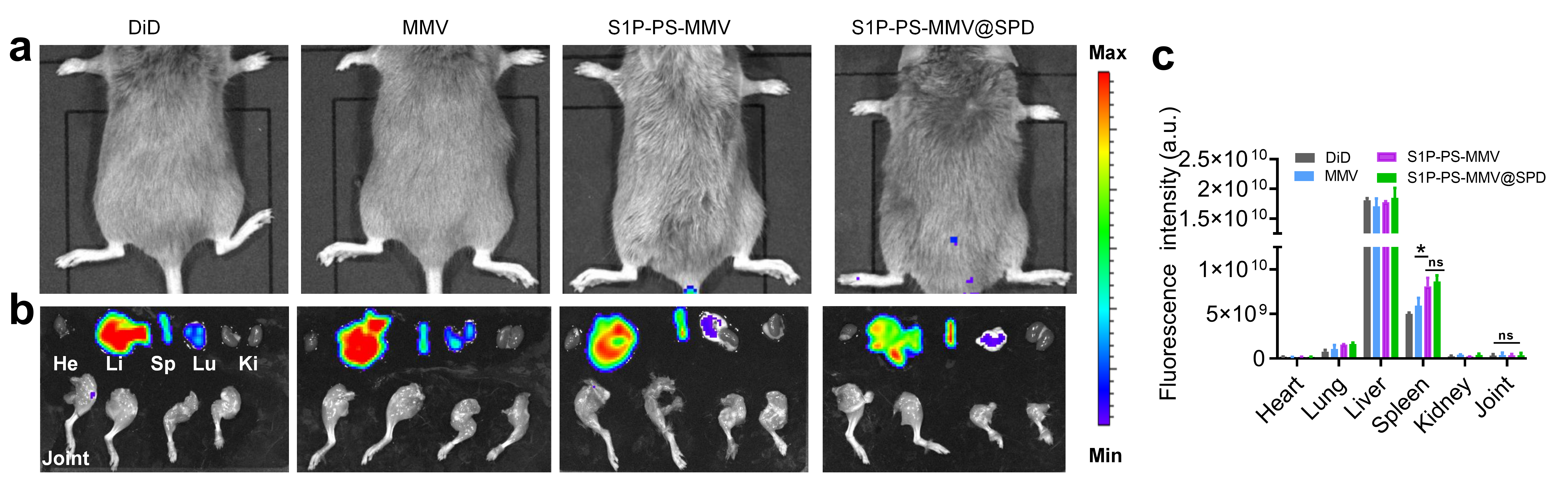


**Figure S13.** Biodistribution of vesicles in normal mice after i.v. injections. (a) Representative live fluorescence images of different vesicles in normal mice at 24 h after injection. (b) Representative fluorescence images and (c) quantitative analysis of fluorescent signals accumulated in main organs and paws at 24 h (d). Data were expressed as mean ± SD (n = 3). ns = no significance.


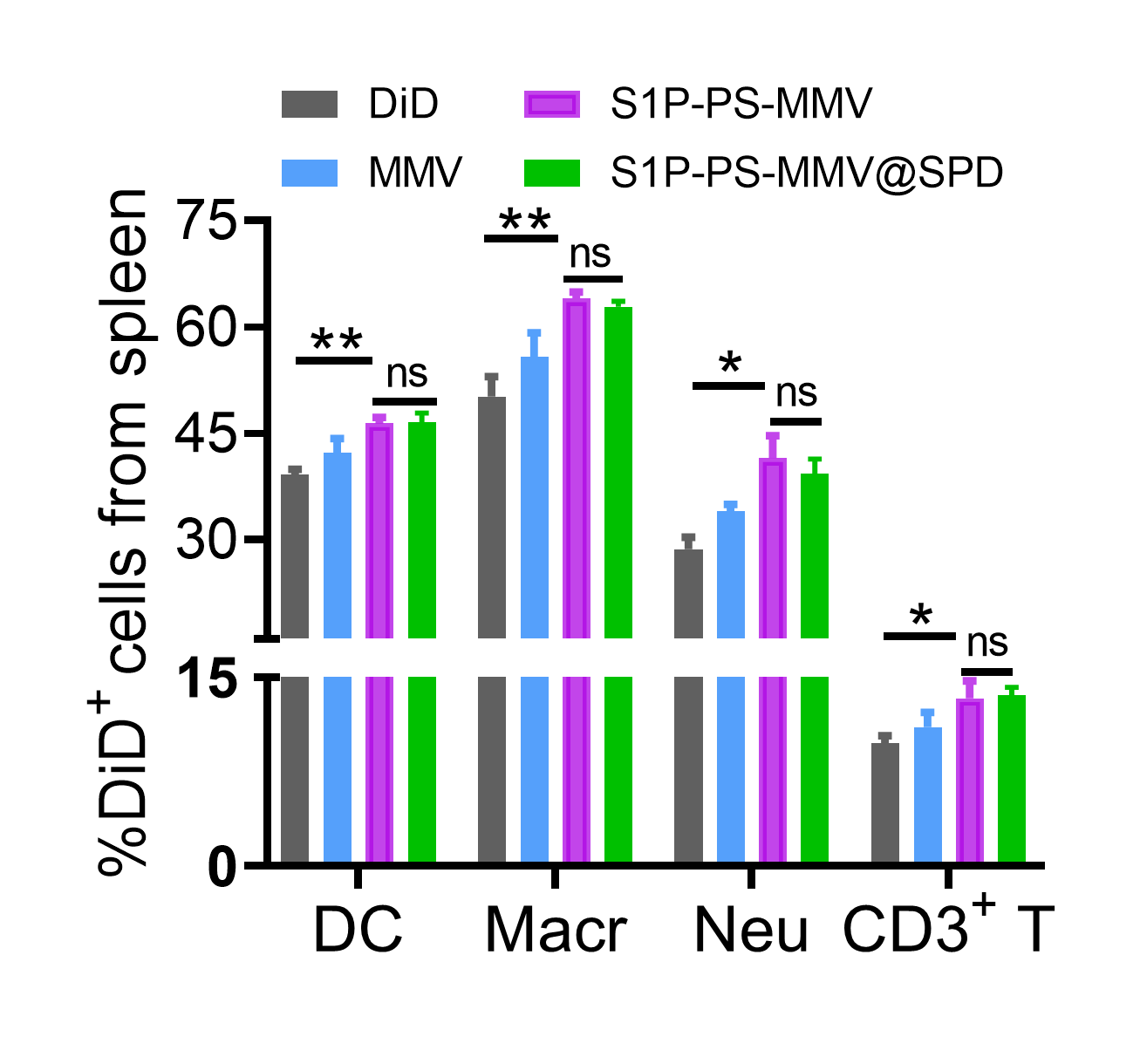


**Figure S14.** Uptake of vesicles by different immune cells in the spleen from normal mice at 24 h after intravenous tail vein injection. DC: dendritic cells, Macr: macrophages, Neu: neutrophils. Data were expressed as mean ± SD (n = 3). ^*^*p* < 0.05, ^**^*p* < 0.01, ns = no significance.


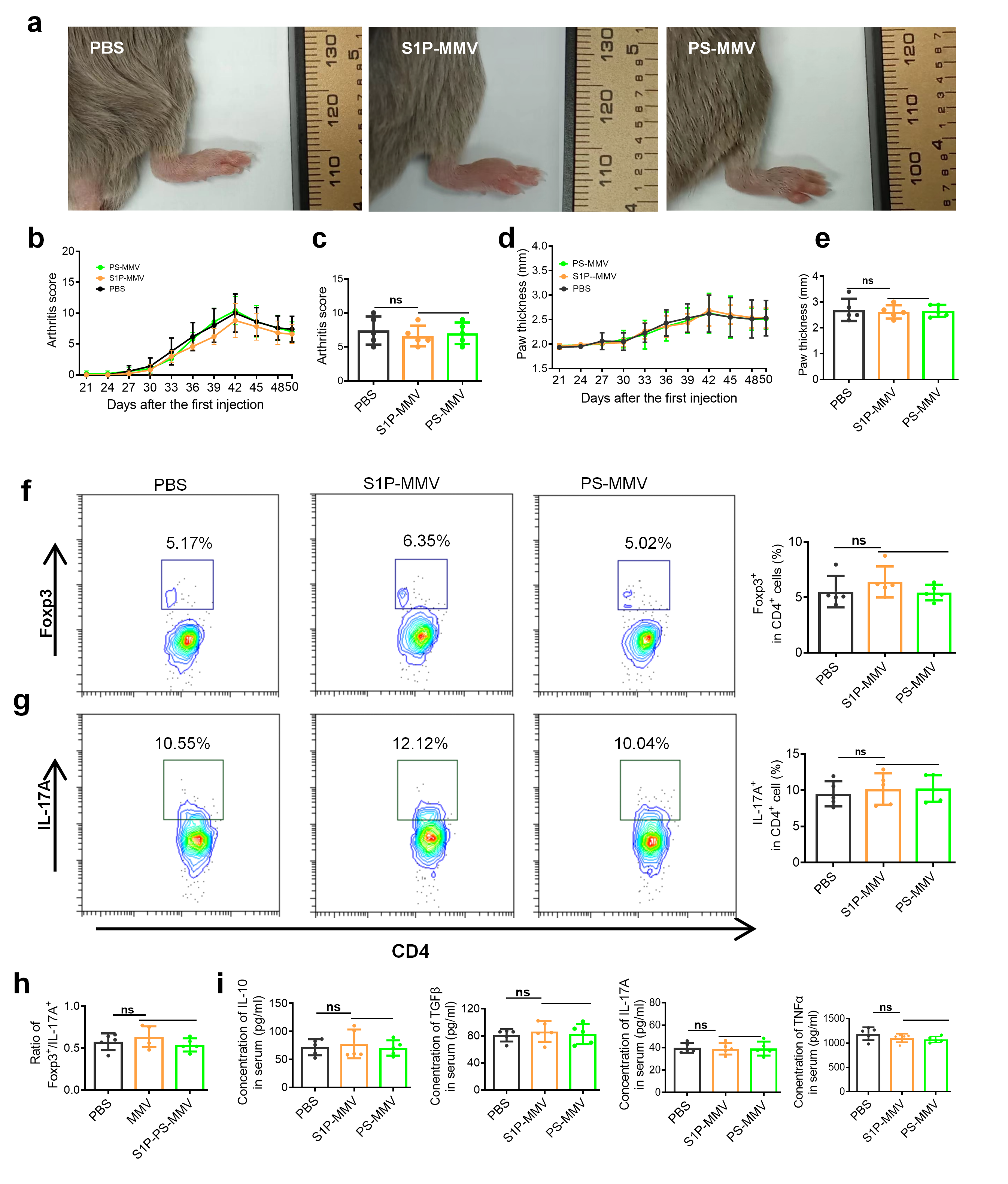


**Figure S15.** The therapeutic effect of vesicles in RA model. (a) Representative macroscopic images of hind paw on day 50 after different treatments. (b) The change of arthritis scores in mice after different treatments and (c) the average of arthritis scores on day 50. (d) The change of hind paw thickness after different treatments and (e) the average of paw thickness on day 50. (f) Representative flow cytometric plots indicated the percentage of CD3^+^CD4^+^Foxp3^+^ Treg and (g) CD3^+^CD4^+^IL-17A^+^ Th17 in spleen of CIA mice after different treatments on day 50. (h) The ratio of Treg/Th17 in spleen of CIA mice after different treatments on day 50. (i) The levels of TNF-α, IL-17, IL-10, and TGF-β in serum of CIA mice after different treatments on day 50. Data are expressed as mean ± SD (n=5). ns = no significance.


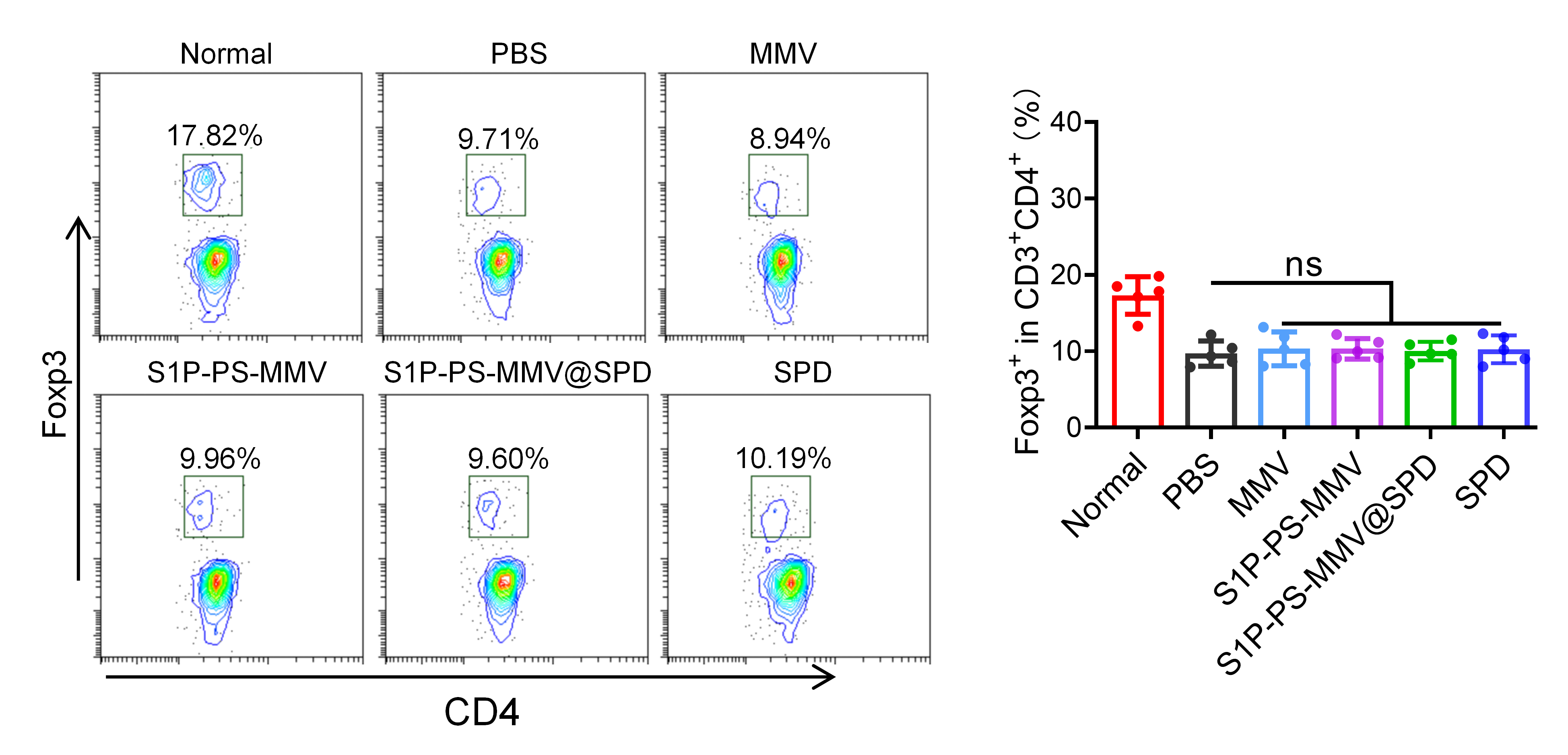


**Figure S16.** The regulatory effects of EMNV on Tre in the lymph nodes *in vivo*. Representative flow cytometric plots indicated the percentage of CD3^+^CD4^+^Foxp3^+^ Treg in the lymph nodes of CIA mice after different treatments on day 50. Data are expressed as mean ± SD (n = 5). ns = no significance.


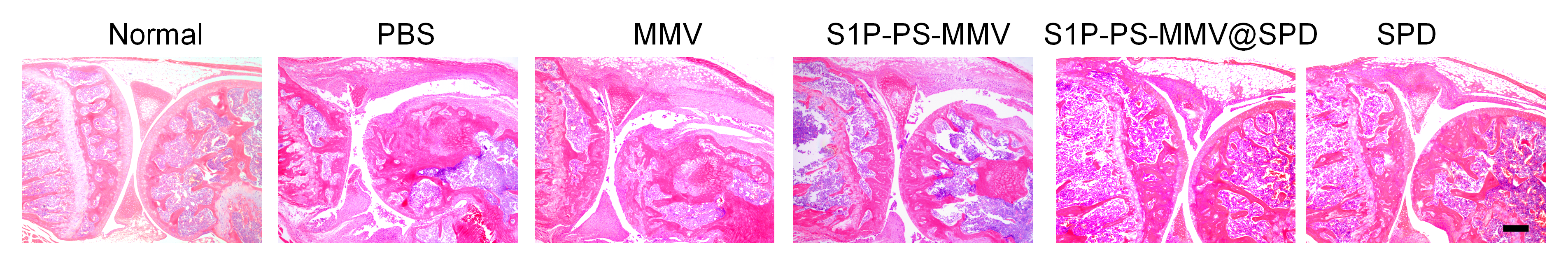


**Figure S17.** Representative H&E staining images in wide-field view of joint tissue sections at the experimental endpoint. Scale bar = 200 µm, the scale bar in the last image also is suitable for the other images.


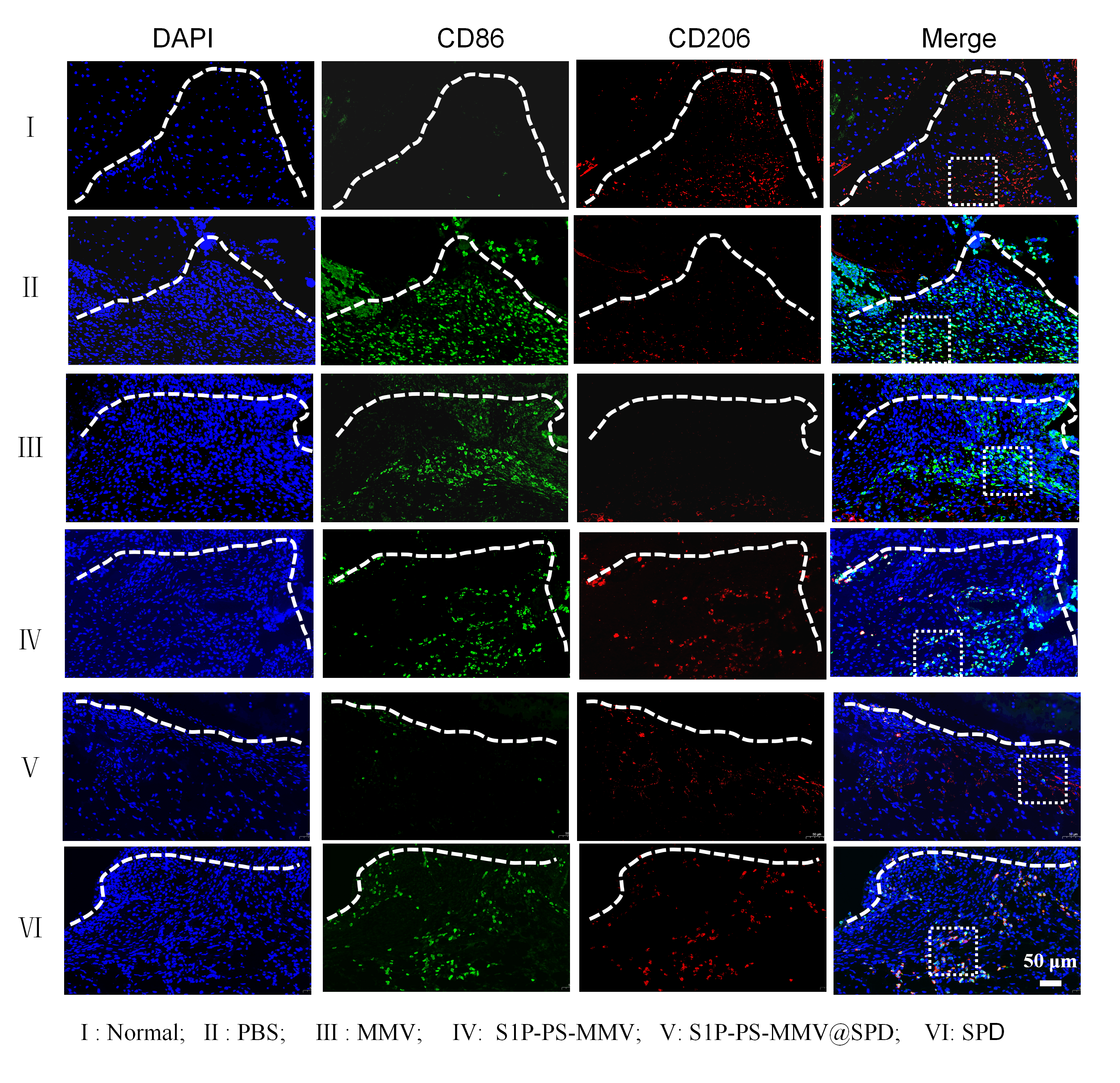


**Figure S18.** Immunostaining images of CD86 and CD206 in wide-field view of joint tissue sections at the experimental endpoint, red: CD206, green: CD86, blue: DAPI, scale bar = 50 µm.


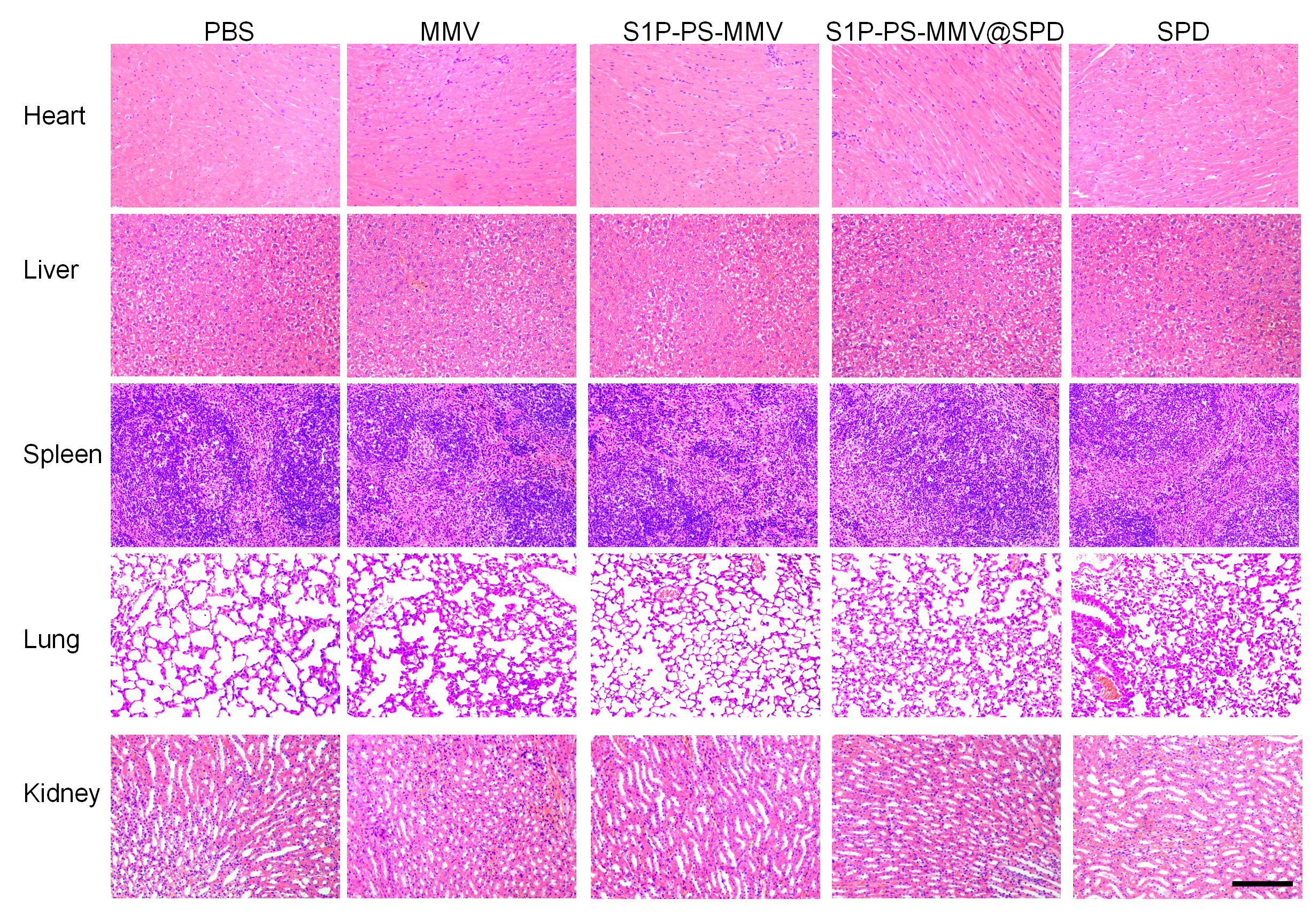


**Figure S19.** H&E staining of major organs in mice after different treatments, scale bar = 200 μm.


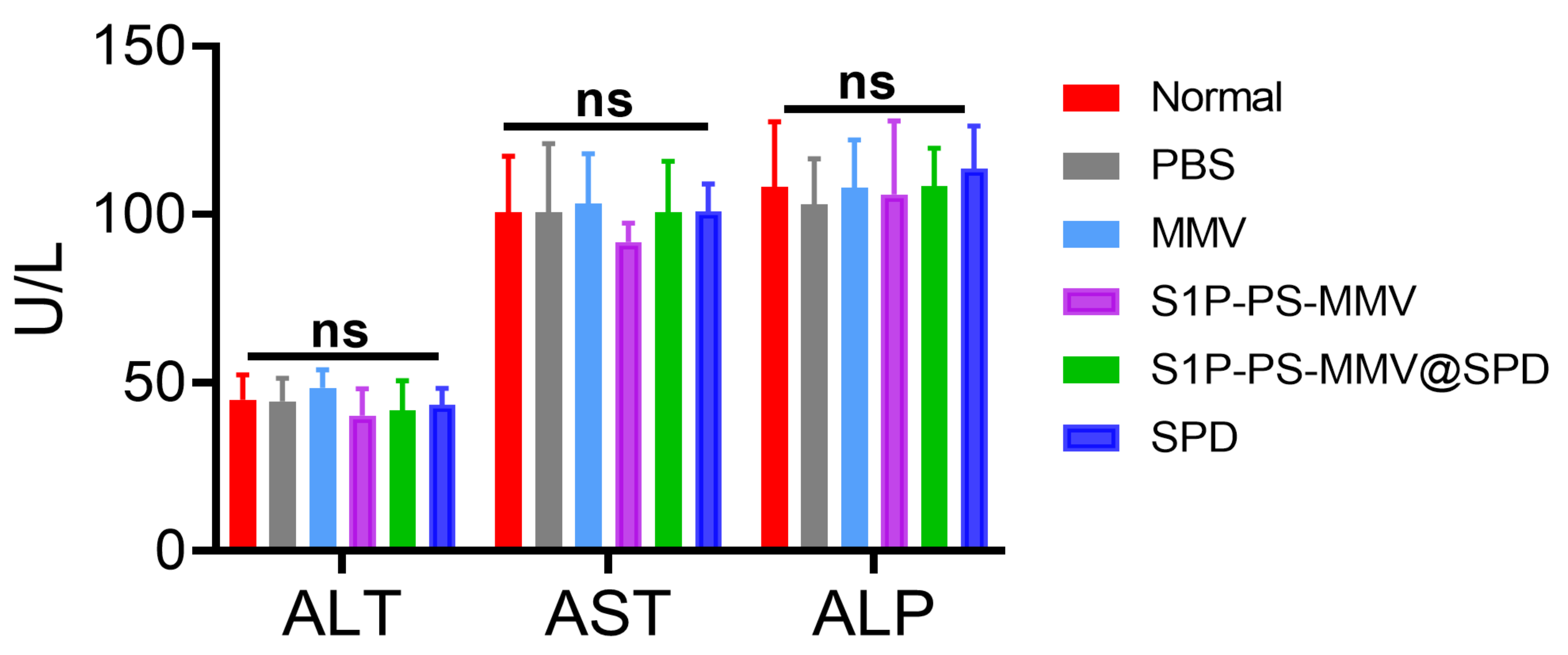


**Figure S20.** The levels of alanine aminotransferase (ALT), alkaline phosphatase (ALP), and aspartate aminotransferase (AST) in the serum after different treaments. Data are expressed as mean ± SD (n=5). ns = no significance.

**Table S1.** Liquid phase gradient elution procedure

| Time (min) | 0 | 10 | 12 | 15 |
| --- | --- | --- | --- | --- |
| Acetonitrile (%) | 10 | 100 | 10 | 10 |
| Ultrapure water (%) | 90 | 0 | 90 | 90 |

**Table S2**. The sequences of primers used for qRT-PCR analysis

| ARG1 | Forward | 5' | TTG GGT GGA TGC TCA CAC TG | 3' |
| --- | --- | --- | --- | --- |
|  | Reverse | 5' | GTA CAC GAT GTC TTT GGC AGA | 3' |
| IL-10 | Forward | 5' | CCAGTACAGCCGGGAAGACA | 3' |
|  | Reverse | 5' | GAAGGCAGTCCGCAGCTCTA | 3' |
| TGFβ | Forward | 5' | ACATAGATCATGCATCTGTCATTTG | 3' |
|  | Reverse | 5' | ATTCTCCTTGGAATCTCACTTCTA | 3' |
| INOS | Forward | 5' | GAGACGCACAGGCAGAGG | 3' |
|  | Reverse | 5' | CAGGCACACGCAATGATGG | 3' |
| IL-6 | Forward | 5' | CTGGAGCCCACCAAGAACGA | 3' |
|  | Reverse | 5' | GCCTCCGACTTGTGAAGTGGT | 3' |
| TNF-α | Forward | 5' | AGGGTCTGGGCCATAGAACT | 3' |
|  | Reverse | 5' | CCACCACGCTCTTCTGTCTAC | 3' |
| MerTK | Forward | 5' | TGGCTTTTGGCGTGACCAT | 3' |
|  | Reverse | 5' | TCCAGCTGCAGCCTCAACAC | 3' |
| ProS | Forward | 5' | CGCCGTGCAAATACCTTGTT | 3' |
|  | Reverse | 5' | AATGAGCCAACACGGAATGC | 3' |
| STAT6 | Forward | 5' | CAGCACTGGGTGCAAAGGGA | 3' |
|  | Reverse | 5' | GGCCCTGGCTCAACACAGTT | 3' |
| PPARγ | Forward | 5' | GCTGGCCTCCCTGATGAATAA | 3' |
|  | Reverse | 5' | TCCCTGGTCATGAATCCTTGG | 3' |
| FOXP3 | Forward | 5' | TTCAGAAACCACCCCGCCAC | 3' |
|  | Reverse | 5' | CCCGCCCACCTTTTCTTGGT | 3' |
| IL-17 | Forward | 5' | CCTGGCGGCTACAGTGAAGG | 3' |
|  | Reverse | 5' | CGGTGGAGAGTCCAGGGTGA | 3' |
| Atg5 | Forward | 5' | AGGCGTGACACCCTTGCTTC | 3' |
|  | Reverse | 5' | GCTGCAGTGGTCCTGTGTGT | 3' |
| LC3 | Forward | 5' | TCTTTTGGGTGCTGGCTGGG | 3' |
|  | Reverse | 5' | GGATGAGGGCAAGATGGGC | 3' |
| GADPH | Forward | 5' | TGTGGATGGCCCCTCTGGAA | 3' |
|  | Reverse | 5' | TGACCTTGCCCACAGCCTTG | 3' |
